# An endogenous green fluorescent protein–photoprotein pair in *Clytia hemisphaerica* eggs shows co-targeting to mitochondria and efficient bioluminescence energy transfer

**DOI:** 10.1098/rsob.130206

**Published:** 2014-04-09

**Authors:** Cécile Fourrage, Karl Swann, Jose Raul Gonzalez Garcia, Anthony K. Campbell, Evelyn Houliston

**Affiliations:** 1 Sorbonne Universités, UPMC Univ Paris 06, Laboratoire de Biologie du Développement de Villefranche-sur-mer (LBDV), Observatoire Océanologique, 06230 Villefranche-sur-mer, France; 2 CNRS, Laboratoire de Biologie du Développement de Villefranche-sur-mer (LBDV), Observatoire Océanologique, 06230 Villefranche-sur-mer, France; 3 School of Medicine, Cardiff University, Cardiff CF14 4XN, UK; 4 School of Pharmacy and Pharmaceutical Sciences, Cardiff University, Redwood Building, King Edward VII Avenue, Cardiff CF10 3NB, UK

**Keywords:** green fluorescent protein, protein coevolution, bioluminescence resonance energy transfer, clytin, Hydrozoa

## Abstract

Green fluorescent proteins (GFPs) and calcium-activated photoproteins of the aequorin/clytin family, now widely used as research tools, were originally isolated from the hydrozoan jellyfish *Aequora victoria*. It is known that bioluminescence resonance energy transfer (BRET) is possible between these proteins to generate flashes of green light, but the native function and significance of this phenomenon is unclear. Using the hydrozoan *Clytia hemisphaerica*, we characterized differential expression of three clytin and four GFP genes in distinct tissues at larva, medusa and polyp stages, corresponding to the major *in vivo* sites of bioluminescence (medusa tentacles and eggs) and fluorescence (these sites plus medusa manubrium, gonad and larval ectoderms). Potential physiological functions at these sites include UV protection of stem cells for fluorescence alone, and prey attraction and camouflaging counter-illumination for bioluminescence. Remarkably, the clytin2 and GFP2 proteins, co-expressed in eggs, show particularly efficient BRET and co-localize to mitochondria, owing to parallel acquisition by the two genes of mitochondrial targeting sequences during hydrozoan evolution. Overall, our results indicate that endogenous GFPs and photoproteins can play diverse roles even within one species and provide a striking and novel example of protein coevolution, which could have facilitated efficient or brighter BRET flashes through mitochondrial compartmentalization.

## 2. Introduction

Both bioluminescence and fluorescence are widespread natural phenomena, particularly in the marine environment [1–3]. Green light flashes in the hydrozoan jellyfish *Aequora victoria* are generated by two famous proteins acting together, the calcium-sensitive photoprotein aequorin and green fluorescent protein (GFP). These proteins and their engineered derivatives are today used for a vast range of applications, including subcellular calcium imaging, cell lineage tracing, gene regulation analysis and detecting protein–protein interactions [4].

The phenomena of bioluminescence and fluorescence are distinct. Bioluminescence involves generation of light from a biochemical reaction, the oxidation of a ‘luciferin’ substrate, catalysed by a photoprotein such as aequorin or by another type of luciferase [5]. Fluorescence involves light emission from a fluorophore following energy absorption, usually from light of a shorter, higher energy wavelength. Confusion can arise because photoproteins themselves can be fluorescent, but also notably because many organisms contain both photoproteins and other fluorescent proteins (FPs), and show coupling of their activity through a radiationless energy transfer process, termed bioluminescence resonance energy transfer (BRET). In the case of *Aequora*, aequorin generates blue light with a broad emission peak centred around a wavelength of 460 nm, which directly excites GFP molecules to be re-emitted as green light with a much sharper peak centred at 508 nm [6–10].

Several distinct families of lucifierases have been characterized and are thought to have arisen independently during evolution [2,11,12]. Aequorin family luciferases are found in cnidarians and ctenophores, although in anthozoan cnidarians their bioluminescence function appears to have been lost [13]. They require calcium ions as essential cofactors and catalyse the oxidation of small, tightly associated, substrate molecules called coelenterazines, which are considered part of the functional photoprotein. Binding of aequorins to Ca^2+^ promotes oxidation of coelenterazine into coelenteramide, and blue light is emitted as this relaxes to the ground state. Aequorin family photoproteins from hydrozoan species include clytins (or phialidins) from the genus *Clytia* (formerly *Phialidium*), obelin from *Obelia* and mitrocomin from *Mitrocoma* (=*Halistaura*), in addition to aequorin from *Aequora* [14–16].

Many FPs related to GFP have also been isolated from cnidarians. Variation in amino acid sequence around the much conserved ‘SYG’ fluorophore site affects absorption and emission spectra, generating a wide range of brightness and colour properties especially among anthozoans. Most hydrozoan species only have one FP (typically green), although cyan and yellow types have been isolated from *Obelia* and from an unidentified *Philalidium* species [17,18]. BRET between aequorin family photoproteins and FPs has been widely demonstrated in cnidarians through spectral studies showing that the *in vivo* bioluminescence precisely matches that of the corresponding purified GFP both in the wavelength and narrowness of the emission peak [7,8,10]. In species lacking GFP, such as the scyphozoan jellyfish *Pelagia* and the ctenophore *Mnemiopsis*, bioluminescence is blue, with characteristics matching that of the purified photoprotein [7]. BRET between the molecules relies on an absorption peak of GFP centred around 475 nm and requires a distance between the molecules of less than 10 nm, which can be achieved by the formation of transient complexes through electrostatic interactions when the two proteins are present at micromolar concentrations [9,19].

Despite the extensive biochemical and structural studies, relatively little attention has been paid to when and where the endogenous aequorins and FP genes are expressed in their species of origin. Historic studies described light-producing organs (‘photophores’) around the rim of the bell in medusae of the hydrozoan species *Aequora forskalea* and *Mitrocoma cellukinia.* These distinctive yellow-pigmented structures flank the tentacle bulb or form a broad continuous line, sandwiched between the ectoderm and endoderm of the circular canal between the tentacle bulbs [20,21], and were later found to fluoresce green under UV illumination. In the considerably smaller medusae of *Clytia gregarium*, tiny light flashes were recorded in the early studies, coinciding with the tentacle bulbs themselves, but no pigmented photophores were distinguishable. An additional dull glow from the gonads was sometimes seen [20]. This could be accounted for by the bioluminescence later described in eggs, in a study also able to record bioluminescence in planula larva and, following metamorphosis, in the polyp form [22].

We have examined the genetic basis of differential fluorescence and bioluminescence in the hydrozoan *Clytia hemisphaerica*, taking advantages of availability of molecular resources and laboratory-reared medusae [23]. We identified multiple GFP and clytin cDNA sequences, characterized their expression in relation to the distribution of endogenous bioluminescence and fluorescence and analysed BRET *in vivo.* Our results shed light on the physiological function of BRET and uncovered an unexpected subcellular compartmentalization of this process in spawned eggs, achieved by coevolution of mitochondrial targeting sequences of a particular GFP–Cyclin gene pair.

## 3. Results

### 3.1. Coelenterazine-dependent bioluminescence in *Clytia* eggs and tentacle bulbs

**Figure 1.**
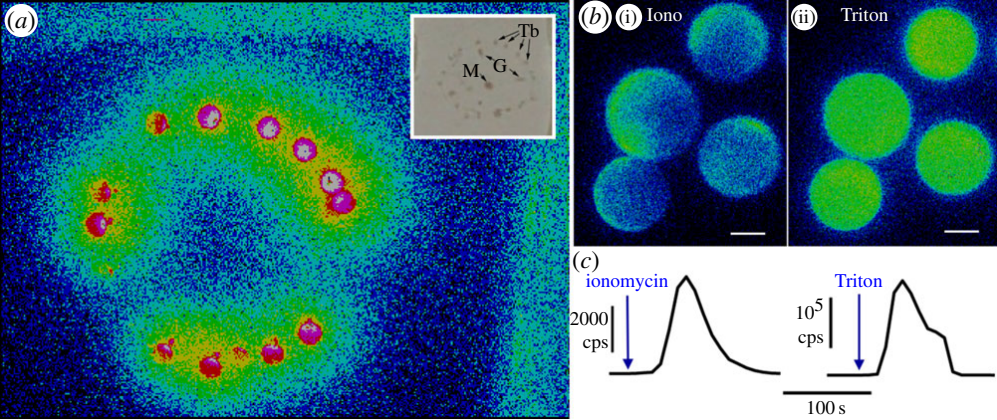
Bioluminescence in *C. hemisphaerica*. Bioluminescence image of an immobilized whole adult medusa, approximately 1 cm in diameter, stimulated with 0.5 M KCl. Pixels are colour coded according to a scale in which hot colours (including white) indicate the highest levels of bioluminescence, and cold colours represent low bioluminescence. (*a*) Inset shows the same medusa by light microscopy indicating the position of tentacle bulbs (Tb), gonads (G) and manubrium (M). (*b*) Eggs (approx. 180 µm in diameter) treated first with ionomycin (*b*(i)) and subsequently treated with Triton X-100 to lyse the egg and discharge all clytin photoprotein luminescence (*b*(ii)). The images show luminescence from 200 s of integrated light collection after ionomycin and then after detergent treatment. Equivalent images taken before the treatment were completely black. Scale bars 100 µm. (*c*) Luminescence kinetics from one of seven similar eggs treated in the same way.

We visualized the sites of bioluminescence in *Clytia hemisphaerica* medusae (figure 1) stimulated by treatment with calcium ionophore, detergents or 0.5 M KCl to cause a rise in cytoplasmic calcium concentration [24]. Bioluminescence was detected at very restricted sites at the base of each of tentacle bulb around the bell margin (figure 1*a*). We cannot rule out the possibility that additional sites emit low-level bioluminescence, undetectable by our methods. As previously shown in *C. gregarium* [22], bioluminescence was detectable in spawned *C. hemisphaerica* eggs (figure 1*b*,*c*) and could be fully discharged by detergent lysis. Importantly, we found that pre-incubation in coelenterazine was required for the detection of bioluminescence in our laboratory-reared *Clytia* medusae and eggs, probably because this essential photoprotein substrate is normally supplied in the marine crustacean diet but not present in the artemia we used for feeding [25,26].

### 3.2. Green fluorescence at multiple sites in *Clytia*

**Figure 2.**
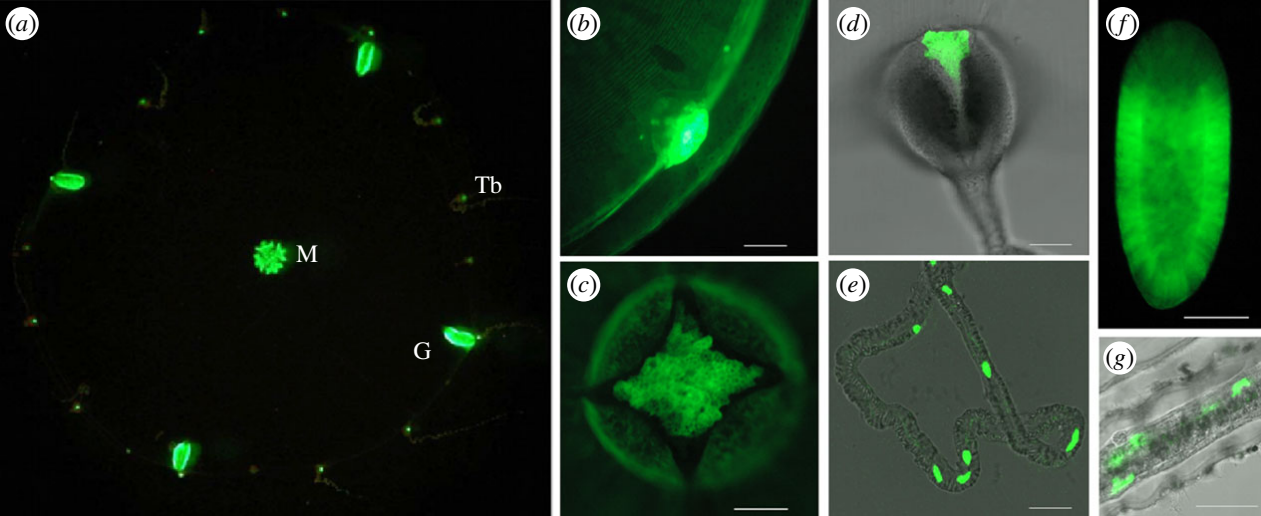
Fluorescence in *C. hemisphaerica.* Green fluorescence observed upon excitation with blue light under a stereomicroscope (*a*), fluorescence microscope (*b*,*f*) or confocal microscope with 488 nm laser excitation (*c*,*d*,*e*,*g*), superimposed on transmitted light images in (*d*,*e*,*g*). (*a*) Adult medusa immobilized in agar (courtesy of A. Amiel); M, manubrium; G, gonad; Tb, tentacle bulb. (*b*) Bell margin with tentacle bulb (baby medusa), (*c*) manubrium (baby medusa), (*d*) tentacle bulb, (*e*) tentacle, (*f*) planula larva (2 days post fertilization), (*g*) stolon. For a guide to medusa organization, see figure 4. All scale bars 100 µm.

The distribution of endogenous green fluorescence in *C. hemisphaerica* was readily detectable by fluorescence microscopy upon excitation with blue light, in the absence of coelenterazine (figure 2). Highly fluorescent structures included both major sites of bioluminescence (tentacle bulb spots and oocytes/eggs) but also two additional sites of fully grown adult jellyfish, the manubrium and the gonad (figure 2*a*,*c*). Closer examination of the tentacle bulb photophore revealed small masses of highly fluorescent cells adjacent to the endoderm, and individual fluorescent cells extending into the central region of each tentacle (figure 2*b*,*d*,*e*). In the manubrium (mouth and stomach region) and gonad, strong green fluorescence was found in both endodermal and ectodermal cells (figure 2*c*). Weaker fluorescence was observed across the bell (figure 2*b*). The planula larva showed strong fluorescence, localized to a subpopulation of epitheliomuscular ectodermal cells, largely absent from the aboral pole region (figure 2*f*). In polyp colonies, green fluorescence was observed in isolated cells in the endoderm of stolon, hydranth (figure 2*g*) and polyp tentacles as well as in some nematocytes (not shown). Fluorescence without bioluminescence in gonad somatic tissue and in planula larvae in *C. hemisphaerica* contrasts with the lack of green fluorescence in these tissues reported in *C. gregarium* [22] but has been reported in the manubrium of *Obelia* medusa [17,27].

### 3.3. Gene expression underlying fluorescence and bioluminescence

Four clearly distinct GFP (CheGFP1–GFP4) and three distinct clytin sequences were identified from our *Clytia* mixed-stage transcriptome collection [23,28]. *Clytia hemisphaerica* clytin1 and clytin2 are orthologues of the previously characterized *C. gregarium* photoproteins clytin-I and clytin-II [15] (confirmed by phylogenetic analysis: see below). The clytin sequences were closely related to each other, 94% similar and 77% identical at the amino acid level, while the CheGFP sequences were 52% similar and 22% identical at the amino acid level (excluding the N terminal leader sequences—see below).

**Figure 3.**
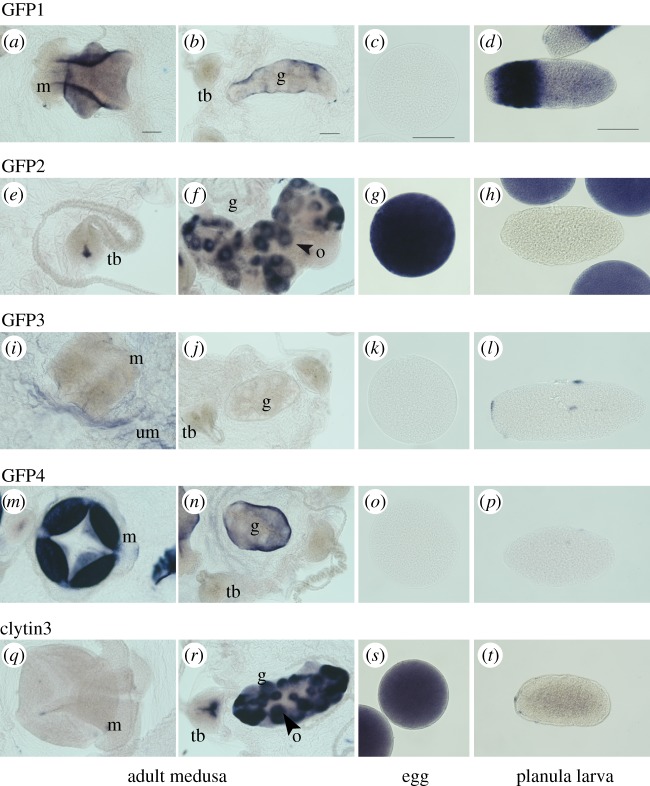
Sites of CheGFP and clytin gene expression. GFP (*a*–*p*) and clytin (*q*–*t*) expression detected by *in situ* hybridization in adult *C. hemisphaerica* medusae, egg and planula larvae. GFP1 is detected in manubrium and gonad ectoderm (*a*,*b*) and in planula larva (*d*). GFP2 expression is strongly detected in unfertilized eggs (*g*), and restricted to oocytes (*f*) and a small proximal zone in tentacle bulb (*e*) in adult female tissues. GFP3 appears weakly expressed in umbrella (*i*), while GFP4 is strongly expressed in manubrium (*m*) and gonad ectoderm (*n*). *In situ* hybridization with a clytin3 antisense probe detects the combined expression of the three clytin genes in a proximal and central zone of tentacle bulbs and oocytes of gonad (*r*), strong expression in eggs (*s*) and weaker expression in planula (*t*). Clytin1 and clytin2 probes gave equivalent results owing to the cross-detection of highly similar sequences. m, manubrium; g, gonad; o, oocyte; tb, tentacle bulb; um, bell. Scale bars, 100 µm.

**Figure 4.**
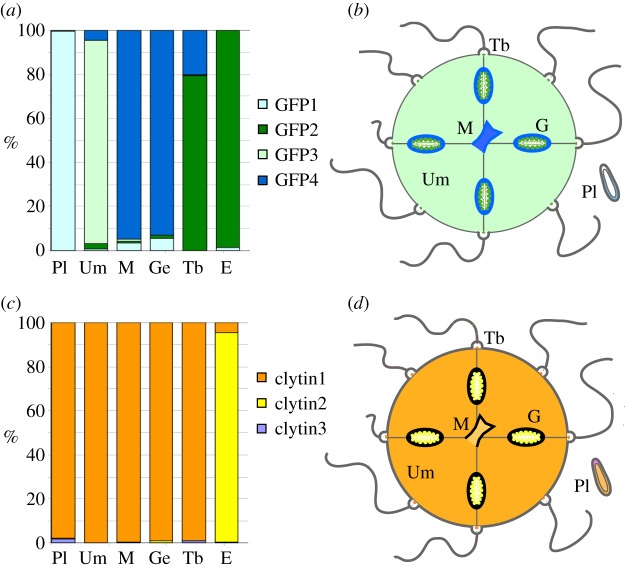
Relative expression of CheGFP and clytin genes evaluated by Q-PCR. (*a*) Q-PCR analysis showing relative expression (% of total) of each GFP gene. (*b*) Schematic summary of GFP differential expression in *Clytia*. (*c*) Relative expression (% of total) of each clytin gene measured by Q-PCR. (*d*) Schematic summary of clytin differential expression in *Clytia*. All Q-PCR primer sequences are provided in the electronic supplementary material, table S1. Annotations for all panels: Pl, planulae; Um, bell; M, manubrium; G, gonad; Ge, gonad ectoderm; Tb, tentacle bulb; E, eggs.

Each of the four *C. hemisphaerica* GFP genes was found to have a distinct stage- and tissue-specific expression profile, revealed by *in situ* hybridization (figure 3) and quantitative PCR (Q-PCR) (figure 4). The individual GFP gene expression profiles (figure 3*a–p*) covered all the sites of green fluorescence, indicating that no significantly expressed GFP genes in the *Clytia* genome had been overlooked. CheGFP1 expression accounted for the fluorescence in the planula larva ectoderm and it is also expressed significantly in the medusa manubrium and gonad ectoderm. CheGFP4 is strongly expressed at the same medusa sites but not in the planula. Expression of CheGFP2, the ‘maternal’ GFP, was strongly detected in developing oocytes as well as in spawned eggs, and also at the tentacle bulb photophores, but not elsewhere. CheGFP3 *in situ* signal was distributed mainly in the thin ectoderm of the medusa umbrella, where some CheGFP4 expression was also apparent.

The strong sequence similarity of the three *C. hemisphaerica* clytin genes (see above) precluded synthesis of specific probes for *in situ* hybridization analysis. The combined clytin expression pattern, obtained using antisense probes to any of the three transcripts (figure 3*q*–*t*), mirrored the sites of bioluminescence (figure 1), with a strong signal observed in oocytes, eggs and the centre of the tentacle bulb. A weak signal was also observed across other medusa tissues, including the manubrium. Expression territories for the individual clytin genes were determined by Q-PCR analysis (figure 4*c*). In all the adult somatic tissue tested, we detected predominantly clytin3 mRNA, while eggs were found to express almost exclusively clytin2. Clytin1 was barely detected in any tissue sample.

We conclude that the selective sites of fluorescence and bioluminescence in *Clytia* result from differential GFP and clytin genes deployment. Bioluminescence is generated by GFP2 combined with clytin3 or clytin2 in tentacles and eggs, respectively, while the strong green fluorescence in the manubrium and gonad ectoderm is generated by GFP4 and GFP1. In the larval ectoderm, GFP1 provides green fluorescence and may also combine with low levels of clytin3 to produce faint bioluminescence. The differential expression of GFP and clytin genes supports the hypothesis that multiple roles for fluorescence and bioluminescence, and possibly other functions for these proteins, coexist in this species.

### 3.4. Bioluminescence energy transfer between clytin and GFP *in vivo*

**Figure 5.**
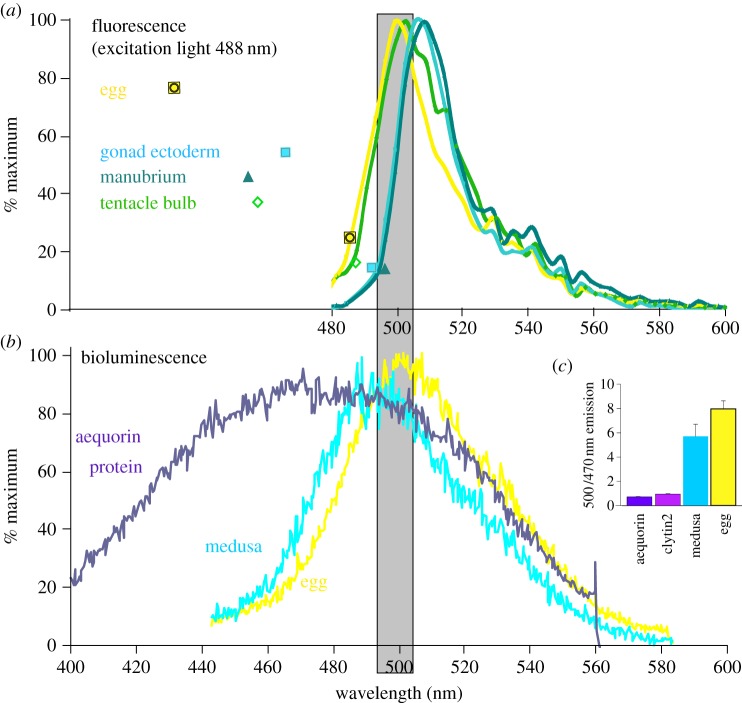
Endogenous luminescence coupling. (*a*) Fluorescence emission spectra for the four GFPs expressed in their endogenous tissues, collected using a Leica SP5 confocal microscope using 488 nm excitation. (*b*) Luminescence spectra obtained for recombined and Ca^2+^-stimulated aequorin compared to a group of eggs and an individual whole adult medusa following detergent stimulation [29]. Multiple spectra obtained from several individual medusae showed very little variation. Aequorin was used in this experiment rather than clytin because it was more easily available but has a very similar emission spectrum [9,15]. (*c*) Light emission ratios (500/470 nm) from recombined and Ca^2+^-stimulated aequorin or clytin2 protein was compared to those from eggs or medusae that had been preloaded with coelenterazine and then lysed with Triton to induce Ca^2+^-dependent luminescence. An average ratio from measurements made on six individual medusae is shown.

To assess the extent of the BRET between clytin and GFP *in vivo*, we determined the GFP fluorescence emission spectra for eggs and selected tissues (figure 5*a*) and the luminescence spectra for groups of whole jellyfish or collected spawned eggs (figure 5*b*).

Fluorescence spectra were determined using a confocal microscope to provide 488 nm wavelength excitation light (see Material and methods; figure 5*a*). The medusa and eggs analysed were all from our laboratory cultures with no exogenous coelenterazine provided, so there was no possibility of bioluminescence under these conditions. Manubrium and gonad tissue showed a fluorescence emission peak at 508–510 nm, whereas in eggs and tentacle bulbs the emission peak lays at 500–502 nm, indicating that maternally expressed CheGFP2 has an emission spectrum distinct from that of CheGFP1 and CheGFP4. The fluorescence spectra obtained may include a slight contribution of fluorescence from the calcium-discharged form of clytin, which emits at 506 nm peak but with excitation maxima at 270 or 345 nm [8]. Unlike in *Obelia* [17] and an undetermined *Clytia* species [18], no yellow or cyan colour variants were detected in *C. hemisphaerica*.

Luminescence spectra were determined following detergent treatment of medusae or eggs to trigger an intracellular Ca^2+^ increase (figure 5*b*). Bioluminescence from eggs and adults was green. The bioluminescence emission spectrum from eggs was significantly sharper and greener than that of purified photoprotein (aequorin included in figure 5*b* for comparison) and directly matched the fluorescence emission spectrum for GFP (compare figure 5*a* (488 nm excitation) and figure 5*b* (bioluminescence): shaded zone), indicative of complete BRET between the molecules [7]. The occurrence of BRET was also tested using our custom-built luminometer, which collected light from 10 nm bands at two wavelengths, 500 and 470 nm (figure 5*c*). This technique confirmed that the bioluminescence of recombinant *C. hemisphaerica* clytin2 protein was indeed blue, as well documented for aequorin and *C. gregarium* clytin [7,9,15], and that *in vivo* bioluminescence from *C. hemisphaerica* eggs is green. Assuming that endogenously expressed clytin2 has the same bioluminescence emission properties as purified clytin2, these findings clearly imply that in eggs BRET between the protein pair clytin2–GFP2 is highly effective.

Overall bioluminescence analysed from whole medusae showed a slightly broader and shorter wavelengthed emission peak than that from eggs (figure 5*b*). Correspondingly, the 500/470 nm ratio measured from whole medusae was slightly lower than that from eggs (figure 5*c*). Thus, while green bioluminescence involving BRET to GFP clearly predominates in medusae, where bioluminescence mainly originates from the GFP-rich tentacle bulb photophores (figure 1*a*), weak bioluminescence from other sites with lower clytin expression and thus less complete BRET could account for the shifted emission spectrum obtained from whole medusae. An equivalent shift can be obtained experimentally *in vitro* by lowering the concentrations of clytin and GFP proteins [8]. Our methods to determine bioluminescence were not sensitive enough to compare emission characteristics between medusae tissues, and we did not determine bioluminescence characteristics in any other life-cycle stages.

### 3.5. Mitochondrial targeting of maternal GFP and clytin proteins

**Figure 6.**
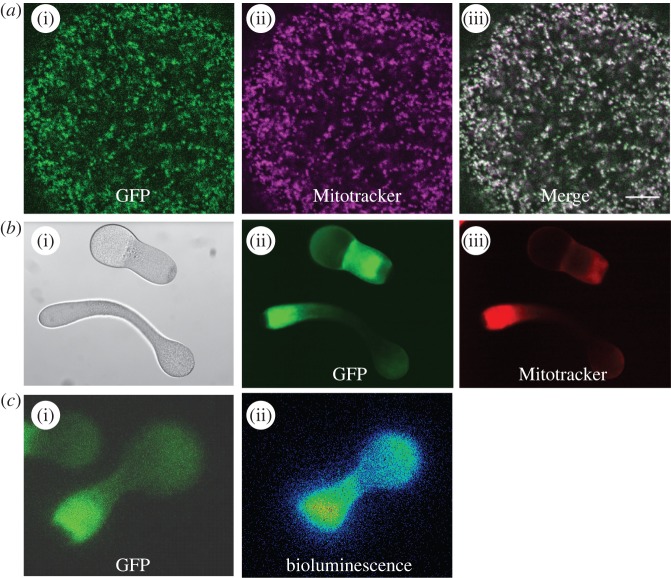
*Clytia* GFP2 and clytin2 are targeted to egg mitochondria. (*a*) Confocal images of eggs labelled with Mitotracker Deep Red. (*a*(i)) GFP fluorescence, (*a*(ii)) Mitotracker, (*a*(iii)) merge. (*b*) Stratified eggs comparing GFP versus Mitotracker orange distribution. (*b*(i)) Brightfield, (*b*(ii)) GFP fluorescence, (*b*(iii)) Mitotracker orange. (*c*) Stratified eggs comparing green fluorescence (*c*(i)) and Triton lysis-induced luminescence (*c*(ii)). All scale bars 100 µm.

The spectral comparisons revealed intriguing high-efficiency *in vivo* coupling between the co-expressed clytin2 and GFP2 proteins in *Clytia* eggs compared with medusa tissues. A further surprise came from closer examination of protein localization in the egg, where we found both fluorescence and bioluminescence localized to mitochondria (figure 6). Firstly, as reported briefly elsewhere [30], confocal microscopy showed the maternally expressed GFP2 protein to be contained within discrete vesicular structures, confirmed as mitochondria by comparison with the vital Mitotracker (figure 6*a*) or TMRE dyes, or use of antibodies recognizing the mitochondrial protein VDAC (not shown). Furthermore, low-speed egg centrifugation co-stratified green fluorescence with organelles stained with Mitotracker Orange (figure 6*b*). In such centrifuged eggs, coelenterazine-dependent, detergent-induced bioluminescence was emitted from the mitochondria-rich zone (figure 6*c*), indicating that the maternal clytin as well as GFP is targeted to mitochondria.

**Figure 7.**
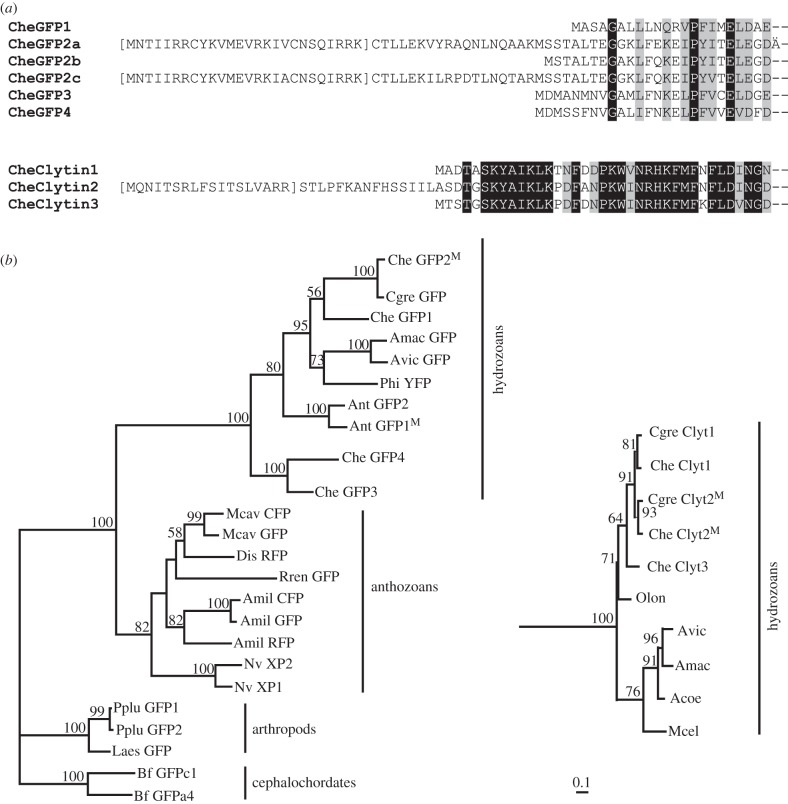
Diversification of GFP and clytin genes. (*a*) Amino acid alignments of the N terminus of deduced *Clytia* GFP and clytin protein sequences. GFP2a, GFP2c and clytin2 sequences show an N terminal presequence, predicted with high probability to mediate targeting to mitochondria (Mitoprot software). Predicted cleaved sequences are shown in brackets. Black shading: identical amino acids; grey shading, similar amino acids. (*b*) Phylogenetic relationships of representative cnidarian *GFP* and *clytin* genes, deduced by ML from the alignments provided in the electronic supplementary material, figure S1. Sequences of arthropod and cephalochordate GFPs were used as an out-group for the GFPs tree (*a*), and sequences of calmodulin protein were used as an out-group for the clytin tree (*b*). Superscript M indicates the presence of a predicted mitochondrial targeting sequence. Bootstrap percentages (500 replicates) over 50% are shown. The scale bar indicates the number of amino acid substitutions per site. Acoe, *Aequorea coerulescens*; Amac, *Aequorea macrodactyla*; Avic, *Aequorea Victoria*; Amil, *Acropora millepora*; Ant, *Anthomedusae* species; Bf, *Branchiostoma floridae*; Cgra, *Clytia gracilis*; Cgre, *Clytia gregarium*; Che, *Clytia hemisphaerica*; Dis, *Discosoma* species; Laes, *Labidocera aestiva*; Mcav, *Montastraea cavernosa*; Mcel, *Mitrocoma cellularia*; Nv, *Nematostella vectensis*; Olon, *Obelia longissima*; Phy, *Phialidium* species; Pplu, *Pontellina plumata*; Rren, *Renilla reniformis.* Sequence accession numbers are given in the electronic supplementary material, table S2.

A further strong indication that GFP2 and clytin2 are targeted to egg mitochondria came from sequence analysis (figure 7). The predicted amino acid sequence of clytin2 and of some CheGFP2 variants displayed N-terminal leaders of 30–50 amino acids, which were shown using Mitoprot (http://ihg.gsf.de/ihg/mitoprot.html) [31] or iPsort (http://ipsort.hgc.jp/) [32] protein localization prediction software to have high probability (more than 0.8) to direct protein targeting and internalization within the mitochondrial lumen. For GFP2, pre-sequences with putative cleavage sites were present in sequences of two of the three variants from our EST collection (figure 7*a*). The three variants were otherwise quasi-identical at the nucleotide level, suggesting that the putative targeting sequence may derive from an alternative spliced N terminal exon. This was confirmed by the analysis of preliminary raw genomic sequence data from the ongoing *C. hemisphaerica* genome sequence project. Concerning clytin2 protein, sequence analysis predicted targeting to mitochondria with 0.9 probability. In contrast to *Clytia* GFP2, no leaderless cDNA variants were identified in our transcriptomic data, while genome sequence analysis confirmed that the 5′ leader sequence to this independent gene is not coded by a separate exon.

Biochemical studies had previously provided evidence that bioluminescence in certain cnidarian and ctenophore species is generated in membrane-bound organelles termed lumisomes, around 0.2 µm in diameter [33,34]. *In vitro* lysis of these particles disrupts BRET, suggesting that they act to maintain high concentrations of photoproteins and GFPs locally. We propose that in the *Clytia* egg, mitochondrial targeting may fulfil an equivalent compartmentalization function (see Discussion).

### 3.6. Evolutionary history of clytin and GFP mitochondrial targeting

Taken together, the expression data, the tight endogenous spectral coupling, N-terminal sequence analysis and colocalization studies strongly suggest that GFP2 and clytin2 function together as a closely linked pair in mitochondria, and may have coevolved during expansion of their respective gene families. To address how this unusual situation for two unrelated genes arose during evolution, we performed phylogenetic analysis of available predicted protein sequences for the two families. GFP genes have been detected in a disparate selection of animals across many phyla, suggesting a complex evolutionary history [35,36]. Duplications have arisen separately within various evolutionary lineages, including copepods and cephalochordates [37,38] as well as in anthozoan and hydrozoan cnidarians. Our phylogenetic analyses showed that within the Hydrozoa, a first duplication probably separated the CheGFP 3 + 4 from the CheGFP 1 + 2 lineages, to which most of the other identified hydrozoan GFP sequences belong (figure 7*b*; sequence alignments in the electronic supplementary material, figure S1). By contrast, the clytins are clearly part of a well-defined monophyletic and phylogenetically restricted group of photoproteins, whose sequence evolution mirrors the relations between hydrozoan families. Aequorin/clytin family proteins are thought to have emerged in a common metazoan ancestor, these genes and/or their bioluminescence function having probably subsequently been lost in the bilaterian, anthozoan and sponge lineages [13].

Searches for mitochondrial targeting predictions among publically available FP and photoprotein sequences revealed a convincing leader in a GFP protein from an undefined hydrozoan jellyfish species (AntGFP1) and in CheGFP2, as well and in *C. hemisphaerica* and *C. gregarium* clytin2 (marked M in figure 7*b*), but not in any other known GFP or aequorin family proteins. Mitochondrial targeting of both families thus appears restricted to the hydrozoans. More widespread occurrence of mitochondrial targeting cannot be ruled out without more comprehensive genomic or transcriptomic data from a greater number of species, but mitochondrial targeting of both families appears to be restricted to the hydrozoans and to have arisen together within this group.

## 4. Discussion

We have undertaken the first systematic study of the deployment of fluorescence and bioluminescence genes in a cnidarian. We have shown that in *C. hemisphaerica* fluorescence and bioluminescence associate with distinct structures and life-cycle stages and that this results from differential expression of multiple GFP and aequorin family photoprotein genes. We propose that gene family expansion and differential expression of the paralogues allowed the acquisition of multiple functions for bioluminescence and fluorescence during hydrozoan evolution. Our findings demonstrate that in an endogenous context the GFP and aequorin proteins can function either separately or in a physiologically coupled manner. The green bioluminescence emitted from *C. hemisphaerica* eggs almost certainly results from effective BRET coupling between the maternally expressed clytin2–GFP2 protein pair. These two proteins are co-targeted to mitochondria through parallel acquisition during the evolution of N-terminal tags to their protein sequences. Below, we discuss possible explanations and implications for these findings.

### 4.1. Differential deployment of GFPs and photoproteins in *Clytia*

The striking differential expression of the four GFP genes in *Clytia* suggests that expansion of this gene family in the hydrozoan lineage was linked to region- and/or stage-specific regulation of expression of the paralogues. This may reflect acquisition of different functions as suggested in the cephalochordates [37] and in the sea anemone (anthozoan cnidarian) *Nematostella vectensis* [39]. In the coral *Seriatopora hystrix*, GFP and cyan fluorescent protein are expressed at different life-cycle stages [40]. Differential expression of FPs has also been observed in the hydrozoan *Obelia*, where yellow, green and cyan fluorescence occur in distinct medusa areas. As we found in *Clytia*, these areas of fluorescence are much more extensive than the bioluminescent photocytes positioned around the bell rim.

What adaptive advantages might have driven this diversification of expression sites and probably of function in the GFPs and clytins? Bioluminescence appears to have arisen independently at least 40 times during evolution, and there is evidence for a variety of roles, for instance in predation, defence and counter-illumination [2]. Light flashes in *Clytia* tentacle bulbs and tentacles seem ideally placed to lure prey. In eggs and early embryos, the other principal bioluminescence sites in *Clytia*, possible roles include distracting predators and camouflage, since C*lytia* species come to the surface at dawn (as we observe in *C. hemisphaerica*) and/or dusk to spawn. Light sparks induced by sporadic external calcium entry and the cytoplasmic calcium increase triggered at fertilization [41] might help disguise the eggs from upward-looking predators, against a twinkling water surface. In a survey of 21 hydrozoan species, only four had bioluminescent eggs while eight showed bioluminescence in the medusa [22]. The adaptive advantage of bioluminescence in eggs may relate to the timing and depth at which spawning occurs, factors that vary widely between closely related species consistent with a scenario of rapid evolution.

The weak bioluminescence in the vital but relatively opaque gonad and mouth regions could also serve a camouflaging role when the medusae come to the surface to spawn. Another possible role for bioluminescence is prey distraction: in *Euphysa* medusa, bioluminescent flashes from the bell endoderm are associated with a protective inversion behaviour when under predator attack [42], which may occur to a less dramatic extent in *Clytia*.

Studies in non-bioluminescent animals such as corals have provided evidence that GFPs without photoprotein partners can confer photoprotection and/or contribute to physiological responses to algal symbionts [43–45]. A possible role for GFP in the manubrium could thus be to provide protection from oxidative stress [46] resulting from contact with and digestion of prey. It has been suggested that GFPs could confer protection against UV radiation by transforming absorbed radiation to higher wavelength/less energetic fluorescence [43]. CheGFP1 and CheGFP4 expression in the gonad ectoderm and in the larval ectoderm are well placed to play such a ‘sunscreen’ role. Both these cell layers enclose proliferating cell populations [47] and germ cell precursors vulnerable to UV damage during surface spawning, embryo development and early larval life, which occur close to the sea surface.

GFP2 in the *Clytia* egg and early embryo may also be photoprotective, more specifically for maternal mitochondrial DNA. While eggs and larvae of other animals including cephalochordates and corals also express GFPs [36,48], *C. gregarium* eggs and somatic gonad cells are curiously bioluminescent but not fluorescent [22]. Reduced fluorescence compared with *C. hemisphaerica* may reflect lower exposure to sunlight due to behavioural and/or environmental differences.

### 4.2. Physiological bioluminescence resonance energy transfer in egg mitochondria

In various hydrozoans, it has been inferred that aequorin/clytin family photoproteins and GFP cooperate in BRET. BRET from aequorins to GFP not only shifts the spectrum of light emission but can increase the bioluminescent reaction yield [49]. In *Clytia* medusae, both coexpression and spectral coupling are largely incomplete, but in eggs we were able to demonstrate near-perfect BRET between clytin2 and GFP2 such that the bioluminescence and fluorescence emission spectra closely matched. This was accompanied by co-targeting of the two proteins to mitochondria. A number of hypotheses can be advanced concerning the significance of this novel and highly intriguing finding. One is that mitochondrial targeting promotes effective bioluminescence at relatively low protein levels by co-compartmentalization, facilitating localized maintenance of both proteins to the micromolar concentrations required for BRET via their transient electrostatic association [10,50]. The second possibility is that mitochondrial localization may increase the intensity and duration of light emission; clytin, as an aequorin family member, is likely to have a light output proportional to at least the square of the Ca^2+^ concentration. In mitochondria, the free Ca^2+^ concentration during stimulation reaches a much higher level than that in the cytosol and the Ca^2+^ response may last much longer [51].

Whichever factor provided the evolutionary drive for bioluminescence of *Clytia* eggs, it is conceivable that GFP accumulation in egg mitochondria arose first, owing to reactive oxygen species (ROS) scavenging and/or UV photoprotection capacities (see above), and that the co-targeting of clytin was secondary. Importing GFP to egg mitochondria may be advantageous to protect maternal mitochondrial DNA from UV damage when the spawned eggs are at the sea surface or from ROS produced significantly in mitochondria [52]. GFP also has the potential to enhance mitochondrial-specific reactions such as proton pumping of the respiratory chain [53], raising interesting parallels between a possible role for GFP in mitochondria and that of the plant pigment chlorophylls in chloroplastids. It should be noted that egg bioluminescence does not necessarily involve BRET to GFP; *C. gregarium* [22] and *Mnemiopsis leidyi* eggs show blue bioluminescence but no green fluorescence [6,12]. *Mnemiopsis* does not contain GFP, but extensive gene duplication has generated at least 10 highly similar photoprotein genes in tandem clusters, hypothesized to favour high protein synthesis levels in the specialized photocyte cells of the adult [13], but also potentially in the egg. Whether *C. gregarium* egg clytin is mitochondrially targeted merits reinvestigation. Low-speed centrifugation did not stratify the bioluminescence of *C. gregarium* [22], however the mitochondrial distribution may not have been affected in the experimental conditions used. The available *C. gregarium* clytin2 cDNA sequence does carry a mitochondrial targeting sequence, but alternative transcript forms may exist as they do in *C. hemisphaerica*. The apparent rareness of mitochondrial targeted GFPs and aequorins within the hydrozoans matches the patchy occurrence of egg bioluminescence.

The deployment of GFP and clytin/aequorin photoprotein families is very diverse among cnidarians and ctenophores. During evolution and species diversification, these molecules have acquired various biochemical properties leading not only to a wide range of colour spectra useful for scientists but probably also to a range of physiological functions. Our observations suggest that the highly complex pattern of fluorescence and bioluminescence resulting from differential GFP and clytin gene expression in *C. hemisphaerica* reflects specialization for different roles within a single animal following gene diversification during hydrozoan evolution. The novel coupled expression and mitochondrial targeting of GFP and clytin in the egg provides a striking case of protein coevolution, which merits further examination in the future.

## 5. Material and methods

### 5.1. Animals

All the *Clytia hemisphaerica* used in this study were obtained from our laboratory cultures, raised entirely on *Artemia* larvae throughout the life cycle [23].

### 5.2. Luminescence detection

*Clytia* eggs or whole medusae were pre-incubated in 20 µM f-coelenterazine [54] in seawater for about 3 h. Eggs loaded with coelenterazine were mounted in seawater on a Zeiss Axiovert S100 microscope equipped with a cooled intensified CCD camera (Photek Ltd) to image luminescence. Low-level fluorescence was imaged upon excitation from a halogen lamp using the same camera using standard FITC filters or a Nikon TiU epifluorescence microscope and Photometrics HQ_2_ CCD camera.

For light quantification, groups of 30–50 eggs in 0.9 ml of seawater in a plastic tube were positioned inside a custom-made dual wavelength luminometer composed of two photon counting module-based photomultiplier tubes (ET Enterprises Ltd) held at right angles to each other. Each tube had either a 470 nm or 500 nm interference filter with a 10 nm bandwidth (Thorlabs Ltd) placed between the test tube and the photomultiplier. Light emission at each wavelength was integrated for 1–10 min after stimulating the eggs by adding 100 µl of seawater containing 1% Triton X-100. Purified clytin and aequorin photoproteins were diluted into 0.9 ml of KCl (120 mM), Hepes (20 mM), EGTA (100 µM) buffer and emission was stimulated by the addition of 100 µl of 10 mM CaCl_2_. Clytin2 protein was expressed in a TNT SP6 wheat germ extract system (Promega) from a cDNA clone in Express 1 vector and recombined with 10 µM coelenterazine in the TNT reaction buffer + 1 mM EGTA for 20 h before assays. Purified aequorin protein (regenerated with coelenterazine) was a kind gift of Dr O. Shimomura.

### 5.3. Microscopy and fluorescence emission spectra determination

*In situ* hybridization was performed as previously [28]. Mitochondria were labelled in live cells by incubation for 5–30 min in 0.1 µM TMRE (tetra-methylrhodamine ethyl ester; Invitrogen) or Mitotracker Deep Red 633 (Molecular Probes) in 0.2 µm Millipore-filtered seawater. Fluorescence emission spectra of *Clytia* tissues and life-cycle stages were obtained without coelenterazine pre-incubation using a Leica SP5 confocal microscope. Measurements were performed on live adult medusae or eggs mounted between slide and coverslip in seawater. These were excited with a 488 nm argon laser line and images acquired at successive 3 nm spaced, 5 nm wide bands between 475 and 625 nm using the *xyλ* scanning mode. Quantification was then performed on several ‘regions of interest’ defined to sample particular structures: manubrium, bell, tentacle bulb, gonad ectoderm and eggs. Data were acquired from multiple regions of two separate medusae with similar spectra obtained for each tissue. Additional fluorescence images were obtained using an Olympus epifluorescence microscope.

### 5.4. *Clytia hemisphaerica* green fluorescent protein and clytin sequences

*Clytia hemisphaerica* cDNA clones containing complete ORFs were retrieved from a mixed-stage cDNA library following identification from our EST collection. For phylogenetic analysis, selected sequences were aligned using ClustalW in BioEdit and corrected by eye. Maximum-likelihood (ML) analysis was performed using PhyML, as described previously [55]. GFP sequences from Arthopoda and Cephalochordata were used as out-groups for the GFP tree, and calcium-binding domain of calmodulin sequences for the aequorin one.

### 5.5. RNA isolation, reverse transcription and quantitative PCR

Eggs, oocytes, planula larvae and tissue samples dissected from adult female medusae were collected in Millipore-filtered seawater. Total RNA was isolated using the RNAqueous-Micro system (Ambion), treated with DNase (Ambion) for 30 min at 37°C and stored at −20°C. Reverse transcription was performed using the Super Script VILO cDNA Synthesis Kit (Invitrogen).

Q-PCR was performed using primer pairs designed to amplify 150–250 bp fragments specific for each GFP and clytin sequence (electronic supplementary material, table S1). Primer specificity was confirmed by PCR on the set of cloned cDNA sequences. An EF1α fragment was amplified in parallel as a measure of tissue quantity [47]. Q-PCR reactions were run in triplicate on cDNA from two independent tissue preparations using the LightCycler480 (Roche) and the 2^−ΔΔCt^ method was used to evaluate relative expression of each gene.

## Supplementary Material

GFP and Aequorin family protein sequence alignments

## Supplementary Material

ESM Table 1

## Supplementary Material

ESM Table 2

## Data accessibility

GenBank accession numbers are given in the electronic supplementary material, table S2, which also lists the protein sequences retrieved by BLAST searches of publicly available protein datasets used in phylogenetic analysis.

Phylogenetic data: Alignment in ECM figure 1, Phylogenetic tree in figure 7*b*.
