# Supplementary material for: An endogenous green fluorescent protein–photoprotein pair in Clytia hemisphaerica eggs shows co-targeting to mitochondria and efficient bioluminescence energy transfer
Source: Open Biol. 2014 Apr 9;4(4):130206. doi: 10.1098/rsob.130206 (PMC4043110; doi:10.1098/rsob.130206)
Supplement: GFP and Aequorin family protein sequence alignments [file rsob130206supp1.doc]

**Electronic Supplemental Material (Fourrage et al)**

**Figure S1 . GFP and Aequorin family protein sequence alignments**

(Black boxes: identical amino acids between all sequences ; Grey boxes : similar amino acids)

**S1a: Complete alignment of GFP sequences.**

**CheGFP1**  ---------------------------------------------------MASAGALLLNQRVPFIMELDAEVNGIRFAVRGKGTGDATTGIIDTKFVCTT---GKLPV

**CheGFP2a**  ---MNTIIRRCYKVMEVRKIVCNSQIRRKCTLLEKVYRAQNLNQAAKMSSTALTEGGKLFEKEIPYITELEGDVEGMKFTIKGEGTGDGTTGLIKSKYICTT---GDLPV

**CheGFP2b**  ------------------------------------------------MSTALTEGAKLFQKEIPYITELEGDVEGMKFTIKGEGTGDGTTGLIKSKYICTT---GDLPV

**CheGFP2c**  ---MNTIIRRCYKVMEVRKIACNSQIRRKCTLLEKILRPDTLNQTARMSSTALTEGGKLFQKEIPYVTELEGDVEGMKFTIKGEGTGDGTTGLIKSKYICTT---GDLPV

**CheGFP3**  -----------------------------------------------MDMANMNVGAMLFNKELPFVCELDGEINGHKFSVRGKGIGNSAQGLTHGIHVCTS---GELPV

**CheGFP4a**  -----------------------------------------------MDMSSFNVGALIFNKELPFVVEVDFDIDDHKFSIRGKGVGNAEKGLMVGKYVVSE---GELPC

**CheGFP4b**  -----------------------------------------------MDMTSFNVGALIFNKELPFVVEVDFDIDDHKFSIRGKGVGNAEKGLMIGKYVVSE---GELPC

**CgreGFP**  -------------------------------------------------MTALTEGAKLFEKEIPYITELEGDVEGMKFIIKGEGTGDATTGTIKAKYICTT---GDLPV

**AvicGFP**  ----------------------------------------------------MSKGEELFTGVVPVLVELDGDVNGQKFSVSGEGEGDATYGKLTLNFICTT---GKLPV

**AmacGFP**  ----------------------------------------------------MSKGEELFTGVVPILVELDGDVHGHKFSVRGEGEGDADYGKLEIKFICTT---GKLPV

**PhiYFP**  ----------------------------------------------------MSSGALLFHGKIPYVVEMEGNVDGHTFSIRGKGYGDASVGKVDAQFICTT---GDVPV

**AntGFP1**  ------------------MNVMRYNRGFCRVLQNGVKNLRSRNCSTEEKPVILGAMTETFQKKLPYKLELDGDVDGQTFKVIGEGVGDATTGVIEGKYVCTE---GEVPI

**AntGFP2**  -------------------------------------------------------MAEYFEKPLPYKVELEGDVDGQKFTVIGEGQGDASTGRVEGKYVCTK---GEVPI

**RrenGFP**  ----------------------------------------------------MDLAKLGLKEVMPTKINLEGLVGDHAFSMEGVGEGNILEGTQEVKISVTK--GAPLPF

**McavGFP**  --------------------------------------------------------MSVIKPDMKIKLRMEGAVNGHNFVIEGEGKGKPFEGTQTINLTVKE--GGPLPF

**McavCFP**  --------------------------------------------------------MSVIKSVMKIKLRMDGIVNGHKFMITGEGEGKPFEGTHTIILKVKE--GGPLPF

**AmilGFP**  ----------------------------------------------------MSYSKQGIVQEMKTKYHMEGSVNGHEFTIEGVGTGYPYEGKQMSELVIIKPAGKPLPF

**AmilRFP**  ----------------------------------------------------MALSKHGLTKDMTMKYHMEGSVDGHKFVITGHGNGNPFEGKQTMNLCVVE--GGPLPF

**AmilCFP**  ----------------------------------------------------MSYSKQGIAQVMKTKYHMEGSVNGHEFTIEGVGTGNPYEGTQMSELVITKPAGKPLPF

**NvXP1**  MFLASVWYSFYQKIFIFVSSKTLLPPPLDYAQRYAMLLRIQASPVVYSRFIAALNEYSLSKDAMKLHFKMEGSVNGHCFEIQGVGEGKAFDGEHWSKLCVVK--GKHLPF

**NvXP2**  --------------------------------------------------------MSLSKDAMKLHLILEGSVNGHCFEIHGEGEGKAFEGEQWSKFTVKK--GGPLPF

**DisRFP**  ----------------------------------------------------MRSSKNVIKEFMRFKVRMEGTVNGHEFEIEGEGEGRPYEGHNTVKLKVTK--GGPLPF

**PpluGFP1**  -----------------------------------------------------------MPA-MKIECRISGTLNGVVFELVGGGEGIPEQGRMTNKMKSTK---GALTF

**PpluGFP2**  -----------------------------------------------------------MPA-MKIECRITGTLNGVEFELVGGGEGTPEQGRMTNKMKSTK---GALTF

**LaesGFP**  -----------------------------------------------------------MPV-MKIECRISGTMNGEEFELVGAGDGNTDEGRMTNKMKSTK---GPLSF

**BfGFPa4**  -----------------------------------------------------------MPLPATHEIHLHGSVNGHEFDLVGGGKGDPKAGSLVTEVKSTK---GPLKF

**BfGFPc1**  -----------------------------------------------------------MPLPTTHEVHVYGSINGVEFDLVGSGKGNPKDGSEEIQVKSTK---GPLGF

**CheGFP1**  PWASISSTMAYGALCFAKYPDSVK--DFFKSAMPDG-YIQEKTISFE-NDGAYKVRGVIT--YEHGSIYNRVTLKGEGFKKDGLILQKQYEFCCPNSAVYVLPDKENNGL

**CheGFP2a**  PWATILSSISYGVFCFAKYPRHIA--DFFKSTQPDG-YSQDRIISFD-DDGQYDVKAKIT--YEDGTLYNRVVLDGTGFKSNGNILGMRVLYHSPPHIIYILPDRKNGGM

**CheGFP2b**  PWATILSSLSYGVFCFAKYPRHIA--DFFKSTQPDG-YSQDRIISFD-DDGQYDVKAKIT--YEDGTLYNRVVLKGTGFKSNGNILGMRVLYHSPPHIIYILPDRKNGGM

**CheGFP2c**  PWATILSSLSYGVFCFAKYPRHIA--DFFKSTQPDG-YSQDRIISFD-DDGQYDVKAKIT--YEDGTLYNRVMLDGTGFKSNGNILGMRVLYHSPPHIIYILPDRKNGGM

**CheGFP3**  SWTAITHNLQYGLLCFSQYPHDIN--DYIKSTFPEG-YTKERTSNFD-GDGKYTSRHVIT--YENGCIYNRVTINGSGFSDDGNVLGKNLADYEKPVCSMYFPGKD--GL

**CheGFP4a**  SWSAITHNFQYGQVCFTRYPKDIP--DHIKSLFPEG-YVQTRHSHFV-DDGEYTSVHTLT--YENGVIYNRVKVNGGGFKPDGNVFGKRLREVEPDICSVYFPGKD--GY

**CheGFP4b**  SWSAITHNFQYGQVCFTRYPKDIP--DHIKSLFPEG-YVQTRHSHFV-DDGEYTSVHTLT--YENGVIYNRVKVNGGGFKPDGNVFGKRLREVEPDICSVYFPGKD--GY

**CgreGFP**  PWATILSSLSYGVFCFAKYPRHIA--DFFKSTQPDG-YSQDRIISFD-NDGQYDVKAKVT--CENGTLYNRVTVKGTGFKSNGNILGMRVLYHSPPHAVYILPDRKNGGM

**AvicGFP**  PWPTLVTTFSYGVQCFSRYPDHMKQHDFFKSAMPEG-YVQERTIFYK-DDGNYKTRAEVK--FEGDTLVNRIELKGIDFKEDGNILGHKMEYNYNSHNVYIMGDKPKNGI

**AmacGFP**  PWPTLVTTLGYGILCFARYPEHMKMNDFFKSAMPEG-YIQERTIFFQ-DDGKYKTRGEVK--FEGDTLVNRIELKGMDFKEDGNILGHKLEYNFNSHNVYIMPDKANNGL

**PhiYFP**  PWSTLVTTLTYGAQCFAKYGPELK--DFYKSCMPEG-YVQERTITFE-GDGVFKTRAEVT--FENGSVYNRVKLNGQGFKKDGHVLGKNLEFNFTPHCLYIWGDQANHGL

**AntGFP1**  SWVSLITSLSYGAKCFVRYPNEIN--DFFKSTFPSG-YHQERKITYE-NDGVLETAAKIT--MESGAIVNRINVKGTGFDKDGHVCQKNLE-SSPPSTTYVVPEGE--GI

**AntGFP2**  SWVSLITSLSYGGKCFVRYPNVIK--DFFKSTFPTG-YHQERKITYE-DDGVLETAAKVT--LESGAIYNRISVKGVGFKKDGNVCKKRLH-SSPPQVSYVVPYGE--GI

**RrenGFP**  AFDIVSVAFSYGNRAYTGYPEEIS--DYFLQSFPEG-FTYERNIRYQ-DGGTAIVKSDIS--LEDGKFIVNVDFKAKDLRRMGPVMQQDIVGMQPS-YESMYTN--VTSV

**McavGFP**  AYDILTAAFQYGNRAFTKYPRDIA--DYFKQSFPEG-YSWERSMTYE-DQGICIIKSDIR--MEGDCFIYEIRYDGVNFPPSGPVMQKKTLKWEPS-TEKMYVR--DGVL

**McavCFP**  AYDILTTAFQYGNRVFTKYPKDIP--DYFKQSFPEG-YSWERSMTFE-DQGVCTVTSDIK--LEGDCFFYEIRFYGVNFPSSGPVMQKKTLKWEPS-TENMYVR--DGVL

**AmilGFP**  SFDILSSVFQYGNRCFTKYPADMP--DYFKQAFPDG-MSYERSFLFE-DGAVATASWNIR--LEGNCFIHKSIFHGVNFPADGPVMKKKTIDWDKS-FEKMTVS--KEVL

**AmilRFP**  SEDILSAAFDYGNRVFTEYPQGMV--DFFKNSCPAG-YTWHRSLLFE-DGAVCTTSADITVSVEENCFYHNSKFHGVNFPADGPVMKKMTTNWEPS-CEKIIPVPRQGIL

**AmilCFP**  SFDILSTVFQYGNRCFTKYPEGMT--DYFKQAFPDG-MSFERSFLYE-DGGVATASWNIR--LERDCFIHKSIYHGVNFPADGPVMKKKTIGWDKA-FEKMTVS--KDVL

**NvXP1**  SFDILMPSMSYGTKQFAKYPAGMT--DFFKAAVENGGLSWERTMTFE-DGGYCTIVNTSE--LKDGSLHYHTNFHGINLKPDGPVMQKRTMGWLPS-VETNIPR--RDTL

**NvXP2**  SFDLIAPCLKYGSKPFVKYPDDMT--DFFKAAVENGGLSWERTMSLEQDGGFCSVVNTSK--LDKDGLHYHMTFQGINLDPNGPVMKKMTMGWLPS-VETNIPR--GNTL

**DisRFP**  AWDILSPQFQYGSKVYVKHPADIP--DYKKLSFPEG-FKWERVMNFE-DGGVVTVTQDPS--LQDGCFIYKVKFIGVNFPSDGPVMQKKTMGWEAS-TERLYPR--DGVL

**PpluGFP1**  SPYLLSHVMGYGFYHFGTYPSGYE--NPFLHAANNGGYTNTRIEKYE-DGGVLHVSFSYR--YEAGRVIGDFKVVGTGFPEDSVIFTDKIIRSNAT-VEHLHPM-GDNVL

**PpluGFP2**  SPYLLSHVMGYGFYHFGTYPSGYE--NPFLHAINNGGYTNTRIEKYE-DGGVLHVSFSYR--YEAGRVIGDFKVVGTGFPEDSVIFTDKIIRSNAT-VEHLHPM-GDNVL

**LaesGFP**  SPYLLSHIMGYGFYHYATFPAGYE--NVYLHAAKNGGYTNTRTERYE-DGGIISVNFTYR--YEGNKVIGDFKVVGSGFPANSVIFTDKIIKSNPT-CEHIYPK-GDNIL

**BfGFPa4**  SPHLMIPHLGYGYYQYLPYPDGPS--PFQTAMLDGSGYKVHRVFNFE-DGGVLSIDYNYS--YEGTHIKSDFKLMGSGFPDDGPVMTSQIVDQDGC-VSKKTYL-NDNTI

**BfGFPc1**  SPYIVVPNIGYGFHQYLPFPDGMS--PFQAAADDGSGYVVHRNIQFE-DGASLTGIYRYS--YDAGHIKGEFRVVGSGFPADGPVMTKSLTAVDWS-VATMLFP-NDTTV

**CheGFP1**  RVVYNTIYKLKDG--GHHLAAHE-QQNTPLGG-GVVDIPNYHHIHAGSIFSKDLEET-RDHMCLVETVRAVNLET-----YN-----

**CheGFP2a**  KIEYNKAFDVMGG--GHQMTRHA-QFNKPLGA-WEEDYPMYHHLSVWTSFGKDPEDDETDHLTIVEVIKAIDLDT-----YK-----

**CheGFP2b**  KIEYNKAFDVMGG--GHQMARHA-QFNKPLGA-WGEDFPMYHHLSVWTSF--DPEDDETDHLTIVEVIKAIDLDT-----YK-----

**CheGFP2c**  KIEYNKAFDVMGG--GHQMTRHA-QFNKPLGA-WEEDYPMYHHLSVWTSFGKDPDDDDTDHLTIVEVIKAIDLET-----YK-----

**CheGFP3**  RAEICKLTETKDG--GYQSCRLEDQVIRPISQ-GPTVPMTHHYNYSSVEYSKDANET-RDHIVMKEITKVSHFIQ------------

**CheGFP4a**  NCEFVKLSETVDG--DYQAIRID-QVIRPLSD-GPSLPMTKLYHHYKFEYSKDANET-REHIIMKEQVHASHHTSK-----------

**CheGFP4b**  NCEFVKLSETVDG--DYQAIRID-QVIRPLSD-GPSLPMTKLYHHYKFEYSKDANET-REHIIMKEQVHASHHTSK-----------

**CgreGFP**  KIEYNKAFDVMGG--GHQMARHA-QFNKPLGA-WEEDYPLYHHLTVWTSFGKDPDDDETDHLNIVEVIKAVDLET-----YR-----

**AvicGFP**  KVNFKIRHNIKDG--SVQLADHY-QQNTPIGD-GPVLLPDNHYLSTQSALSKDPNEK-RDHMILLEFVTAARITHGMDELYK-----

**AmacGFP**  KVNFKIRHNIEGG--GVQLADHY-QTNVPLGD-GPVLIPINHYLSTQTAISKDRNET-RDHMVFLEFFSACGHTHGMDELYK-----

**PhiYFP**  KSAFKIMHEITGSKEDFIVADHT-QMNTPIGG-GPVHVPEYHHITYHVTLSKDVTDH-RDNMSLVETVRAVDCRK----TYL-----

**AntGFP1**  RIIYRNIYPTKDG--HYVVADTQ-QVNRPIRAQGTSAIPTYHHIKSKVDLSTDPEEN-KDHIIIKETNCAFDADFS-----------

**AntGFP2**  RVLYSNIYPTKDG--GYVVADTR-QVNRPIKAEGKSAIPKYHYIKSKIDLSTDPNER-KDHIIIKEVNVASGIDFS-----------

**RrenGFP**  IGECIIAFKLQTG--KHFTYHMRTVYKSKKPVET---MPLYHFIQHRLVKTNVDTASG--YVVQHETAIAAHSTIKKIEGSLP----

**McavGFP**  KGEVNMALLLEGG--GHYRCDFRSTYKAKKRVQ----LPDYHFVDHRIEILSHDNDYN--TVKLSENAEARYSMLPRQAK-------

**McavCFP**  LGDVSRTLLLEGN--KHHRCNFRSTYRAKKGVV----LPEYHFVDHRIEILSHDKDYN--TVEVYENAVARPSMLPIKAK-------

**AmilGFP**  RGDVTMFLMLEGG--GSHRCQFHSTYKTEK-PVT---LPPNHVVEHQIVRTDLGQSAKGFTVKLEAHAAAHVNPLKVK---------

**AmilRFP**  KGDIAMYLLLKDG--GRYRCQFDTIYKAKSDPKE---MPEWHFIQHKLTREDR-SDAKNQKWQLVEHAVASRSALPG----------

**AmilCFP**  RGDVTEFLMLEGG--GYHSCQFHSTYKPEK-PVT---LPPNHVVEHHIVRTDLGQTAKGFTVKLEEHAAAHVNPLKVQ---------

**NvXP1**  LGDINMLLKVNDG--SFLRVQFETVYRFMKPVPAGFKMPPHHFMAYRLTRVDNDEHCD--TVIQHEWSEAFSCFLPEKPTMQLPKLK

**NvXP2**  VGDINMLLKVNDG--SFLRVKFVTVYRFMKPVPAGFKMPPHHFIAFKLTRVENDADCN--VVLLHEWGKAFSCFLPENPTLQLPKLK

**DisRFP**  KGEIHKALKLKDG--GHYLVEFKTIYMAKKPVQ----LPGYYYVDSKLDITSHNKDYT--IVEQYERTEGRHHLFLKAELGSNVGER

**PpluGFP1**  VGSFARTFSLRDG--GYYSFVVDSHMHFKSAIHP--SILQNGGSMFAFRRVEELHSNT--ELGIVEYQHAFKTPTAFA---------

**PpluGFP2**  VGSFARTFSLRDG--GYYSFVVDSHMHFKSAIHP--SILQNGGPMFAFRRVEELHSNT--ELGIVEYQHAFKTPIAFA---------

**LaesGFP**  VNAYTRTWMLRDG--GYYSAQVNNHLHFKTAMHP--TMLQNGGSMFTYRKVEELHSQS--DVGIVEYQHVFKTPTAFA---------

**BfGFPa4**  VDSFDWSYNLQNG--KRYRARVTSNYIFGKPLAA--DVMKKQ-PVFVYRKCYVKSTKT--EITLDEREKAFYELA------------

**BfGFPc1**  VSTIDWTCPTTSG--KRYHATVRTNYTFAKPIAG--SILQKQ-PMFVFRKTEVKASDS--EINLKESQKAFHDLV------------

**S1b: Alignment of GFP sequences corrected by eye for phylogenetic analysis.**

**CheGFP1**  VPFIMELDAEVNGIRFAVRGKGTGDATTGIIDTKFVCTTGKLPVPWASISSTMAYGALCFAKYPDSVKDFFKSAMPDGYIQEKTISFENDGAYKVRGVITYEHGSIYNRV

**CheGFP2a**  IPYITELEGDVEGMKFTIKGEGTGDGTTGLIKSKYICTTGDLPVPWATILSSISYGVFCFAKYPRHIADFFKSTQPDGYSQDRIISFDDDGQYDVKAKITYEDGTLYNRV

**CheGFP2b**  IPYITELEGDVEGMKFTIKGEGTGDGTTGLIKSKYICTTGDLPVPWATILSSLSYGVFCFAKYPRHIADFFKSTQPDGYSQDRIISFDDDGQYDVKAKITYEDGTLYNRV

**CheGFP2c**  IPYVTELEGDVEGMKFTIKGEGTGDGTTGLIKSKYICTTGDLPVPWATILSSLSYGVFCFAKYPRHIADFFKSTQPDGYSQDRIISFDDDGQYDVKAKITYEDGTLYNRV

**CheGFP3**  LPFVCELDGEINGHKFSVRGKGIGNSAQGLTHGIHVCTSGELPVSWTAITHNLQYGLLCFSQYPHDINDYIKSTFPEGYTKERTSNFDGDGKYTSRHVITYENGCIYNRV

**CheGFP4a**  LPFVVEVDFDIDDHKFSIRGKGVGNAEKGLMVGKYVVSEGELPCSWSAITHNFQYGQVCFTRYPKDIPDHIKSLFPEGYVQTRHSHFVDDGEYTSVHTLTYENGVIYNRV

**CheGFP4b**  LPFVVEVDFDIDDHKFSIRGKGVGNAEKGLMIGKYVVSEGELPCSWSAITHNFQYGQVCFTRYPKDIPDHIKSLFPEGYVQTRHSHFVDDGEYTSVHTLTYENGVIYNRV

**CgreGFP**  IPYITELEGDVEGMKFIIKGEGTGDATTGTIKAKYICTTGDLPVPWATILSSLSYGVFCFAKYPRHIADFFKSTQPDGYSQDRIISFDNDGQYDVKAKVTCENGTLYNRV

**AvicGFP**  VPVLVELDGDVNGQKFSVSGEGEGDATYGKLTLNFICTTGKLPVPWPTLVTTFSYGVQCFSRYPDHMKDFFKSAMPEGYVQERTIFYKDDGNYKTRAEVKFEGDTLVNRI

**AmacGFP**  VPILVELDGDVHGHKFSVRGEGEGDADYGKLEIKFICTTGKLPVPWPTLVTTLGYGILCFARYPEHMKDFFKSAMPEGYIQERTIFFQDDGKYKTRGEVKFEGDTLVNRI

**PhiYFP**  IPYVVEMEGNVDGHTFSIRGKGYGDASVGKVDAQFICTTGDVPVPWSTLVTTLTYGAQCFAKYGPELKDFYKSCMPEGYVQERTITFEGDGVFKTRAEVTFENGSVYNRV

**AntGFP1**  LPYKLELDGDVDGQTFKVIGEGVGDATTGVIEGKYVCTEGEVPISWVSLITSLSYGAKCFVRYPNEINDFFKSTFPSGYHQERKITYENDGVLETAAKITMESGAIVNRI

**AntGFP2**  LPYKVELEGDVDGQKFTVIGEGQGDASTGRVEGKYVCTKGEVPISWVSLITSLSYGGKCFVRYPNVIKDFFKSTFPTGYHQERKITYEDDGVLETAAKVTLESGAIYNRI

**RrenGFP**  MPTKINLEGLVGDHAFSMEGVGEGNILEGTQEVKISVTKAPLPFAFDIVSVAFSYGNRAYTGYPEEISDYFLQSFPEGFTYERNIRYQDGGTAIVKSDISLEDGKFIVNV

**McavGFP**  MKIKLRMEGAVNGHNFVIEGEGKGKPFEGTQTINLTVKEGPLPFAYDILTAAFQYGNRAFTKYPRDIADYFKQSFPEGYSWERSMTYEDQGICIIKSDIRMEGDCFIYEI

**McavCFP**  MKIKLRMDGIVNGHKFMITGEGEGKPFEGTHTIILKVKEGPLPFAYDILTTAFQYGNRVFTKYPKDIPDYFKQSFPEGYSWERSMTFEDQGVCTVTSDIKLEGDCFFYEI

**AmilGFP**  MKTKYHMEGSVNGHEFTIEGVGTGYPYEGKQMSELVIIKKPLPFSFDILSSVFQYGNRCFTKYPADMPDYFKQAFPDGMSYERSFLFEDGAVATASWNIRLEGNCFIHKS

**AmilRFP**  MTMKYHMEGSVDGHKFVITGHGNGNPFEGKQTMNLCVVEGPLPFSEDILSAAFDYGNRVFTEYPQGMVDFFKNSCPAGYTWHRSLLFEDGAVCTTSADITVEENCFYHNS

**AmilCFP**  MKTKYHMEGSVNGHEFTIEGVGTGNPYEGTQMSELVITKKPLPFSFDILSTVFQYGNRCFTKYPEGMTDYFKQAFPDGMSFERSFLYEDGGVATASWNIRLERDCFIHKS

**NvXP1**  MKLHFKMEGSVNGHCFEIQGVGEGKAFDGEHWSKLCVVKKHLPFSFDILMPSMSYGTKQFAKYPAGMTDFFKAAVENGLSWERTMTFEDGGYCTIVNTSELKDGSLHYHT

**NvXP2**  MKLHLILEGSVNGHCFEIHGEGEGKAFEGEQWSKFTVKKGPLPFSFDLIAPCLKYGSKPFVKYPDDMTDFFKAAVENGLSWERTMSLEDGGFCSVVNTSKLDKDGLHYHM

**DisRFP**  MRFKVRMEGTVNGHEFEIEGEGEGRPYEGHNTVKLKVTKGPLPFAWDILSPQFQYGSKVYVKHPADIPDYKKLSFPEGFKWERVMNFEDGGVVTVTQDPSLQDGCFIYKV

**PpluGFP1**  MKIECRISGTLNGVVFELVGGGEGIPEQGRMTNKMKSTKGALTFSPYLLSHVMGYGFYHFGTYPSGYENPFLHAANNGYTNTRIEKYEDGGVLHVSFSYRYEAGRVIGDF

**PpluGFP2**  MKIECRITGTLNGVEFELVGGGEGTPEQGRMTNKMKSTKGALTFSPYLLSHVMGYGFYHFGTYPSGYENPFLHAINNGYTNTRIEKYEDGGVLHVSFSYRYEAGRVIGDF

**LaesGFP**  MKIECRISGTMNGEEFELVGAGDGNTDEGRMTNKMKSTKGPLSFSPYLLSHIMGYGFYHYATFPAGYENVYLHAAKNGYTNTRTERYEDGGIISVNFTYRYEGNKVIGDF

**BfGFPa4**  ATHEIHLHGSVNGHEFDLVGGGKGDPKAGSLVTEVKSTKGPLKFSPHLMIPHLGYGYYQYLPYPDGPSPFQTAMLDGSYKVHRVFNFEDGGVLSIDYNYSYEGTHIKSDF

**BfGFPc1**  TTHEVHVYGSINGVEFDLVGSGKGNPKDGSEEIQVKSTKGPLGFSPYIVVPNIGYGFHQYLPFPDGMSPFQAAADDGSYVVHRNIQFEDGASLTGIYRYSYDAGHIKGEF

**CheGFP1**  TLKGEGFKKDGLILQKQYECCPNAVYVLPDGLRVVYNTIYKLKDGGHHLAAHEQQNTPLGGIPNYHHIHAGSIFDLEETHMCLVETVRAVNLET

**CheGFP2a**  VLDGTGFKSNGNILGMRVLHSPPIIYILPDGMKIEYNKAFDVMGGGHQMTRHAQFNKPLGAYPMYHHLSVWTSFDPEDDHLTIVEVIKAIDLDT

**CheGFP2b**  VLKGTGFKSNGNILGMRVLHSPPIIYILPDGMKIEYNKAFDVMGGGHQMARHAQFNKPLGAFPMYHHLSVWTSFDPEDDHLTIVEVIKAIDLDT

**CheGFP2c**  MLDGTGFKSNGNILGMRVLHSPPIIYILPDGMKIEYNKAFDVMGGGHQMTRHAQFNKPLGAYPMYHHLSVWTSFDPDDDHLTIVEVIKAIDLET

**CheGFP3**  TINGSGFSDDGNVLGKNLAYEKPCSMYFPGGLRAEICKLTETKDGGYQSCRLEQVIRPISQPMTHHYNYSSVEYDANETHIVMKEITKVSHFIQ

**CheGFP4a**  KVNGGGFKPDGNVFGKRLRVEPDCSVYFPGGYNCEFVKLSETVDGDYQAIRIDQVIRPLSDPMTKLYHHYKFEYDANETHIIMKEQVHASHHTS

**CheGFP4b**  KVNGGGFKPDGNVFGKRLRVEPDCSVYFPGGYNCEFVKLSETVDGDYQAIRIDQVIRPLSDPMTKLYHHYKFEYDANETHIIMKEQVHASHHTS

**CgreGFP**  TVKGTGFKSNGNILGMRVLHSPPAVYILPDGMKIEYNKAFDVMGGGHQMARHAQFNKPLGAYPLYHHLTVWTSFDPDDDHLNIVEVIKAVDLET

**AvicGFP**  ELKGIDFKEDGNILGHKMENYNSNVYIMGDGIKVNFKIRHNIKDGSVQLADHYQQNTPIGDLPDNHYLSTQSALDPNEKHMILLEFVTAARITH

**AmacGFP**  ELKGMDFKEDGNILGHKLENFNSNVYIMPDGLKVNFKIRHNIEGGGVQLADHYQTNVPLGDIPINHYLSTQTAIDRNETHMVFLEFFSACGHTH

**PhiYFP**  KLNGQGFKKDGHVLGKNLENFTPCLYIWGDGLKSAFKIMHEITGSDFIVADHTQMNTPIGGVPEYHHITYHVTLDVTDHNMSLVETVRAVDCRK

**AntGFP1**  NVKGTGFDKDGHVCQKNLESSPPTTYVVPEGIRIIYRNIYPTKDGHYVVADTQQVNRPIRAIPTYHHIKSKVDLDPEENHIIIKETNCAFDADF

**AntGFP2**  SVKGVGFKKDGNVCKKRLHSSPPVSYVVPYGIRVLYSNIYPTKDGGYVVADTRQVNRPIKAIPKYHYIKSKIDLDPNERHIIIKEVNVASGIDF

**RrenGFP**  DFKAKDLRRMGPVMQQDIVMQPSYESMYTNSVIGECIIAFKLQTGKHFTYHMRVYKSKKPVMPLYHFIQHRLVKVDTASYVVQHETAIAAHSTI

**McavGFP**  RYDGVNFPPSGPVMQKKTLWEPSTEKMYVRVLKGEVNMALLLEGGGHYRCDFRTYKAKKRVLPDYHFVDHRIEIHDNDYTVKLSENAEARYSML

**McavCFP**  RFYGVNFPSSGPVMQKKTLWEPSTENMYVRVLLGDVSRTLLLEGNKHHRCNFRTYRAKKGVLPEYHFVDHRIEIHDKDYTVEVYENAVARPSML

**AmilGFP**  IFHGVNFPADGPVMKKKTIWDKSFEKMTVSVLRGDVTMFLMLEGGGSHRCQFHTYKTEK-PLPPNHVVEHQIVRLGQSATVKLEAHAAAHVNPL

**AmilRFP**  KFHGVNFPADGPVMKKMTTWEPSCEKIIPVILKGDIAMYLLLKDGGRYRCQFDIYKAKSDPMPEWHFIQHKLTRR-SDAKWQLVEHAVASRSAL

**AmilCFP**  IYHGVNFPADGPVMKKKTIWDKAFEKMTVSVLRGDVTEFLMLEGGGYHSCQFHTYKPEK-PLPPNHVVEHHIVRLGQTATVKLEEHAAAHVNPL

**NvXP1**  NFHGINLKPDGPVMQKRTMWLPSVETNIPRTLLGDINMLLKVNDGSFLRVQFEVYRFMKPVMPPHHFMAYRLTRNDEHCTVIQHEWSEAFSCFL

**NvXP2**  TFQGINLDPNGPVMKKMTMWLPSVETNIPRTLVGDINMLLKVNDGSFLRVKFVVYRFMKPVMPPHHFIAFKLTRNDADCVVLLHEWGKAFSCFL

**DisRFP**  KFIGVNFPSDGPVMQKKTMWEASTERLYPRVLKGEIHKALKLKDGGHYLVEFKIYMAKKPVLPGYYYVDSKLDIHNKDYIVEQYERTEGRHHLF

**PpluGFP1**  KVVGTGFPEDSVIFTDKIISNATVEHLHPMVLVGSFARTFSLRDGGYYSFVVDHMHFKSAIILQNGGSMFAFRRELHSNELGIVEYQHAFKTPT

**PpluGFP2**  KVVGTGFPEDSVIFTDKIISNATVEHLHPMVLVGSFARTFSLRDGGYYSFVVDHMHFKSAIILQNGGPMFAFRRELHSNELGIVEYQHAFKTPI

**LaesGFP**  KVVGSGFPANSVIFTDKIISNPTCEHIYPKILVNAYTRTWMLRDGGYYSAQVNHLHFKTAMMLQNGGSMFTYRKELHSQDVGIVEYQHVFKTPT

**BfGFPa4**  KLMGSGFPDDGPVMTSQIVQDGCVSKKTYLTIVDSFDWSYNLQNGKRYRARVTNYIFGKPLVMKKQ-PVFVYRKVKSTKEITLDEREKAFYELA

**BfGFPc1**  RVVGSGFPADGPVMTKSLTVDWSVATMLFPTVVSTIDWTCPTTSGKRYHATVRNYTFAKPIILQKQ-PMFVFRKVKASDEINLKESQKAFHDLV

**S1c: Complete alignment of Photoprotein and Calmodulin sequences.**

**CheClyt1**  ---------------------------------MADTASKYAIKLKTNFDDPKWVNRHKFMFNFLDINGNGKITLDEIVSKASDDICAKLEATPAQTQRHQDAVEAFFKK

**CheClyt2**  MQNITSRLFSITSLVARRSTLPFKANFHSSIILASDTGSKYAIKLKPDFANPKWINRHKFMFNFLDINGDGKITLDEIVSKASDDICAKLDATPEQTQRHQDAIEAFFKK

**CheClyt3**  ---------------------------------MTSTGSKYAIKLKPDFDNPKWINRHKFMFKFLDVNGDGRITLNEIVSKASDDICVKLGATKEQTERHQKAVEAFFRE

**CgreClyt1**  ---------------------------------MADTASKYAVKLRPNFDNPKWVNRHKFMFNFLDINGDGKITLDEIVSKASDDICAKLGATPEQTKRHQDAVEAFFKK

**CgreClyt2**  MLWFTNRLLSMSALAAR-SRLQRTANFHTS--ILLATDSKYAVKLDPDFANPKWINRHKFMFNFLDINGNGKITLDEIVSKASDDICAKLDATPEQTKRHQDAIEAFFKK

**AcoeAeq**  ------------------------------------MTSKYAVKLETDFDNPKWIGRHKHMFNFLDVNKNGKITLNEMVYKASDIVLNNLGATPEQAQRHKEAVEAFFGG

**AmacApo**  ------------------------------------MTSKYAVKLEPDFENPKWVGRHKHMFKFLDVNQNGKISLDEMVYKASDIVINNLGATPEQAKRHKDAVEAFFGG

**AvicAeq**  --------------------------------MLYDVPDYASLKLTSDFDNPRWIGRHKHMFNFLDVNHNGKISLDEMVYKASDIVINNLGATPEQAKRHKDAVEAFFGG

**AvicAeq2**  ------------------------------------------------------------MFNFLDVNHNGRISLDEMVYKASDIVINNLGATPEQAKRHKDAVEAFFGG

**AvicApo**  -----------------------------------MTSEQYSVKLTPDFNNPKWIGRHKHMFNFLDVNHNGRISLDEMVYKASDIVINNLGATPEQAKRHKDAVEAFFGG

**OlonObe**  ------------------------------------MSSKYAVKLKTDFDNPRWIKRHKHMFDFLDINGNGKITLDEIVSKASDDICAKLEATPEQTKRHQVCVEAFFRG

**McelMitro**  ----------------------------------MSMGSRYAVKLTTDFDNPKWIARHKHMFNFLDINSNGQINLNEMVHKASNIICKKLGATEEQTKRHQKCVEDFFGG

**RrenLBP**  -------------------------------------------PEVTASERAYHLRKMKTRMKRVDVTGDGFISR-EDYELIAVRIAKIAKLSAEKAEETRQEFLRVADQ

**CgraCaM**  --------------------------------------------------------------------------------------MRSLGQNPTEAE-----LQDMINE

**CheCaM**  --------------------------------------------------------------------------------------MRSLGQNPTEAE-----LQDMINE

**OlonCaM**  --------------------------------------------------------------------------------------MRSLGQNPTEAE-----LQDMINE

**RrenCaM**  ---------------------------------------------MADQLTEEQIAEFKEAFSLFDKDGDGTITTKELG-----TVMRSLGQNPTEAE-----LQDMINE

**NvCaM**  ---------------------------------------------MADQLTEEQIAEFKEAFSLFDKDGDGTITTKELG-----TVMRSLGQNPTEAE-----LQDMINE

**CheClyt1**  IGMDYGKEVEFPQFVEGWKNLAKHDLKLWSQNKKSLIRDWGEAVFDIFDKDGSGSISLDEWKAYGRISGICPSDEDAEKTFKHCDLDNSGKLDVDEMTRQHLGFWYTLDP

**CheClyt2**  MGMDYGKEVEFPEFVKGWEELAKHDLKLWSQNKSTLIRDWGDAVFDIFDKDGSGSISLDEWKAYGRISGICPSDEDAERTFEICDLDNSGKLDVDEMTRQHLGFWYTLDS

**CheClyt3**  AGMEYDREVAFPAYLEGWKRLATNDLKKWSQNKTTLIRDWGDAVFDIFDKDGSGSISLDEWKAYGRISGICPSDEDAERTFKHCDLDNSGKLDVDEMTRQHLGFWYTLDS

**CgreClyt1**  IGMDYGKEVEFPAFVDGWKELANHDLKLWSQNKKSLIRDWGEAVFDIFDKDGSGSISLDEWKAYGRISGICSSDEDAEKTFKHCDLDNSGKLDVDEMTRQHLGFWYTLDP

**CgreClyt2**  MGMDYGKEVPFPEFIKGWEELAKHDLELWSQNKSTLIREWGDAVFDIFDKDASGSISLDEWKAYGRISGICPSDEDAEKTFKHCDLDNSGKLDVDEMTRQHLGFWYTLDP

**AcoeAeq**  AGLKYDVETEWPEYIEGWKRLSKSELEKWSKNQITLVRLWGDALFDIIDKDQNGAISLDEWKAYTKSAGIIHSSEDCEETFRVCDLDDSGRLDVDEMTRQHLGFWYSMDP

**AmacApo**  AGMKYGVETEWPEYIEGWKNLARTELDRFAKNQITLIRLWGDALFDIIDKDQNGAITLDEWKKYTLSAGIIQSAEDCEITFKVCDLDDSGRLDADEMTRQHIGFWYTMDP

**AvicAeq**  AGMKYGVETDWPAYIEGWKKLATDELEKYAKNEPTLIRIWGDALFDIVDKDQNGAITLDEWKAYTKAAGIIQSSEDCEETFRVCDIDESGQLDVDEMTRQHLGFWYTMDP

**AvicAeq2**  AGMKYGVETDWPAYIEGWKKLATDELEKYAKNEPTLIRIWGDALFDIVDKDQNGAITLDEWKAYTKAAGIIQSSEDCEETFRVCDIDESGQLDVDEMTRQHLGFWYTMDP

**AvicApo**  AGMKYGVETEWPEYIEGWKRLATEELERYSKNQITLIRLWGDALFDIIDKDQNGAITLDEWKAYTKSAGIIQSSEDCEETFRVCDIDESGQLDVDEMTRQHLGFWYTMDP

**OlonObe**  CGMEYGKEIAFPQFLDGWKQLATSELKKWARNEPTLIREWGDAVFDIFDKDGSGTITLDEWKAYGKISGISPSQEDCEATFRHCDLDNSGDLDVDEMTRQHLGFWYTLDP

**McelMitro**  AGLEYDKDTTWPEYIEGWKRLAKTELERHSKNQVTLIRLWGDALFDIIDKDRNGSVSLDEWIQYTHCAGIQQSRGQCEATFAHCDLDGDGKLDVDEMTRQHLGFWYSVDP

**RrenLBP**  LGLAPGVRISVEEAAVN----ATDSLLKMKAEEKAMAVIQSLIMYDCIDTDKDGYVSLPEFKAFLQAVGPDITDDKAITCFNTLDFNKNGQISRDEFLVTVNDFLFGLEE

**CgraCaM**  VDADGNGTIDFPEFLT---MMARKMKDTDSEEE-------IKEAFRVFDKDGNGFISAAELRHVMTNLGEKLTDEEVDEMIREADIDG----------------------

**CheCaM**  VDADGNGTIDFPEFLT---MMARKMKDTDSEEE-------IKEAFRVFDKDGNGFISAAELRHVMTNLGEKLTDE-----------------------------------

**OlonCaM**  VDADGNGTIDFPEFLT---MMARKMKDTDSEEE-------IKEAFRVFDKDGNGFISAAELRHVMTNLGEKLTDEEVDEMIREADIDGDGQVNYEG--------------

**RrenCaM**  VDADGDGTIDFPEFLT---MMARKMKDTDSEEE-------IREAFRVFDKDGDGFISAAELRHVMTNLGEKLTDEEVDEMIREADIDGDGQVNYEEFVKMMTSK------

**NvCaM**  VDADGNGTIDFPEFLT---MMARKMKDTDSEEE-------IREAFRVFDKDGNGFISAAELRHVMTNLGEKLTDEEVDEMIREADIDGDGQVNYEEFVKMMTSK------

**CheClyt1**  NADG--LYGNFV--

**CheClyt2**  NADG--LYGDFVP-

**CheClyt3**  NADG--LYGNFVP-

**CgreClyt1**  NADG--LYGNFVP-

**CgreClyt2**  TSDG--LYGNFVP-

**AcoeAeq**  GCEN--VYGGAVP-

**AmacApo**  ACEK--LYGGAVP-

**AvicAeq**  ACEK--LYGGAVP-

**AvicAeq2**  --------------

**AvicApo**  ACEK--LYGGAVP-

**OlonObe**  EADG--LYGNGVP-

**McelMitro**  TCEG--LYGGAVPY

**RrenLBP**  TALANAFYGDLL--

**CgraCaM**  --------------

**CheCaM**  --------------

**OlonCaM**  --------------

**RrenCaM**  --------------

**NvCaM**  --------------

**S1d: Alignment of Clytin and Clamodulin sequences corrected by eye for phylogenetic analysis.**

**CheClyt1**  MFNFLDINGNGKITLDEIVSKASDDICAKLEATPAQTQRHQDAVEAFFKKIGMDYGKEVEFPQFVEGWKNLAKHDLKLWSQNKKSLIRDWGEAVFDIFDKDGSGSISLDE

**CheClyt2**  MFNFLDINGDGKITLDEIVSKASDDICAKLDATPEQTQRHQDAIEAFFKKMGMDYGKEVEFPEFVKGWEELAKHDLKLWSQNKSTLIRDWGDAVFDIFDKDGSGSISLDE

**CheClyt3**  MFKFLDVNGDGRITLNEIVSKASDDICVKLGATKEQTERHQKAVEAFFREAGMEYDREVAFPAYLEGWKRLATNDLKKWSQNKTTLIRDWGDAVFDIFDKDGSGSISLDE

**CgreClyt1**  MFNFLDINGDGKITLDEIVSKASDDICAKLGATPEQTKRHQDAVEAFFKKIGMDYGKEVEFPAFVDGWKELANHDLKLWSQNKKSLIRDWGEAVFDIFDKDGSGSISLDE

**CgreClyt2**  MFNFLDINGNGKITLDEIVSKASDDICAKLDATPEQTKRHQDAIEAFFKKMGMDYGKEVPFPEFIKGWEELAKHDLELWSQNKSTLIREWGDAVFDIFDKDASGSISLDE

**AcoeAeq**  MFNFLDVNKNGKITLNEMVYKASDIVLNNLGATPEQAQRHKEAVEAFFGGAGLKYDVETEWPEYIEGWKRLSKSELEKWSKNQITLVRLWGDALFDIIDKDQNGAISLDE

**AmacApo**  MFKFLDVNQNGKISLDEMVYKASDIVINNLGATPEQAKRHKDAVEAFFGGAGMKYGVETEWPEYIEGWKNLARTELDRFAKNQITLIRLWGDALFDIIDKDQNGAITLDE

**AvicAeq**  MFNFLDVNHNGKISLDEMVYKASDIVINNLGATPEQAKRHKDAVEAFFGGAGMKYGVETDWPAYIEGWKKLATDELEKYAKNEPTLIRIWGDALFDIVDKDQNGAITLDE

**AvicAeq2**  MFNFLDVNHNGRISLDEMVYKASDIVINNLGATPEQAKRHKDAVEAFFGGAGMKYGVETDWPAYIEGWKKLATDELEKYAKNEPTLIRIWGDALFDIVDKDQNGAITLDE

**AvicApo**  MFNFLDVNHNGRISLDEMVYKASDIVINNLGATPEQAKRHKDAVEAFFGGAGMKYGVETEWPEYIEGWKRLATEELERYSKNQITLIRLWGDALFDIIDKDQNGAITLDE

**OlonObe**  MFDFLDINGNGKITLDEIVSKASDDICAKLEATPEQTKRHQVCVEAFFRGCGMEYGKEIAFPQFLDGWKQLATSELKKWARNEPTLIREWGDAVFDIFDKDGSGTITLDE

**McelMitro**  MFNFLDINSNGQINLNEMVHKASNIICKKLGATEEQTKRHQKCVEDFFGGAGLEYDKDTTWPEYIEGWKRLAKTELERHSKNQVTLIRLWGDALFDIIDKDRNGSVSLDE

**RrenLBP**  RMKRVDVTGDGFISR-EDYELIAVRIAKIAKLSAEKAEETRQEFLRVADQLGLAPGVRISVEEAAVN----ATDSLLKMKAEEKAMAVIQSLIMYDCIDTDKDGYVSLPE

**CgraCaM**  --------------------------MRSLGQNPTEAE-----LQDMINEVDADGNGTIDFPEFLT---MMARKMKDTDSEEE-------IKEAFRVFDKDGNGFISAAE

**CheCaM**  --------------------------MRSLGQNPTEAE-----LQDMINEVDADGNGTIDFPEFLT---MMARKMKDTDSEEE-------IKEAFRVFDKDGNGFISAAE

**OlonCaM**  --------------------------MRSLGQNPTEAE-----LQDMINEVDADGNGTIDFPEFLT---MMARKMKDTDSEEE-------IKEAFRVFDKDGNGFISAAE

**RrenCaM**  AFSLFDKDGDGTITTKELG-----TVMRSLGQNPTEAE-----LQDMINEVDADGDGTIDFPEFLT---MMARKMKDTDSEEE-------IREAFRVFDKDGDGFISAAE

**NvCaM**  AFSLFDKDGDGTITTKELG-----TVMRSLGQNPTEAE-----LQDMINEVDADGNGTIDFPEFLT---MMARKMKDTDSEEE-------IREAFRVFDKDGNGFISAAE

**CheClyt1**  WKAYGRISGICPSDEDAEKTFKHCDLDNSGKLDVDEMTRQHLGFWYTLDPNADGLYGNFV--

**CheClyt2**  WKAYGRISGICPSDEDAERTFEICDLDNSGKLDVDEMTRQHLGFWYTLDSNADGLYGDFVP-

**CheClyt3**  WKAYGRISGICPSDEDAERTFKHCDLDNSGKLDVDEMTRQHLGFWYTLDSNADGLYGNFVP-

**CgreClyt1**  WKAYGRISGICSSDEDAEKTFKHCDLDNSGKLDVDEMTRQHLGFWYTLDPNADGLYGNFVP-

**CgreClyt2**  WKAYGRISGICPSDEDAEKTFKHCDLDNSGKLDVDEMTRQHLGFWYTLDPTSDGLYGNFVP-

**AcoeAeq**  WKAYTKSAGIIHSSEDCEETFRVCDLDDSGRLDVDEMTRQHLGFWYSMDPGCENVYGGAVP-

**AmacApo**  WKKYTLSAGIIQSAEDCEITFKVCDLDDSGRLDADEMTRQHIGFWYTMDPACEKLYGGAVP-

**AvicAeq**  WKAYTKAAGIIQSSEDCEETFRVCDIDESGQLDVDEMTRQHLGFWYTMDPACEKLYGGAVP-

**AvicAeq2**  WKAYTKAAGIIQSSEDCEETFRVCDIDESGQLDVDEMTRQHLGFWYTMDP------------

**AvicApo**  WKAYTKSAGIIQSSEDCEETFRVCDIDESGQLDVDEMTRQHLGFWYTMDPACEKLYGGAVP-

**OlonObe**  WKAYGKISGISPSQEDCEATFRHCDLDNSGDLDVDEMTRQHLGFWYTLDPEADGLYGNGVP-

**McelMitro**  WIQYTHCAGIQQSRGQCEATFAHCDLDGDGKLDVDEMTRQHLGFWYSVDPTCEGLYGGAVPY

**RrenLBP**  FKAFLQAVGPDITDDKAITCFNTLDFNKNGQISRDEFLVTVNDFLFGLEETALAFYGDLL--

**CgraCaM**  LRHVMTNLGEKLTDEEVDEMIREADIDG----------------------------------

**CheCaM**  LRHVMTNLGEKLTDE-----------------------------------------------

**OlonCaM**  LRHVMTNLGEKLTDEEVDEMIREADIDGDGQVNYEG--------------------------

**RrenCaM**  LRHVMTNLGEKLTDEEVDEMIREADIDGDGQVNYEEFVKMMTSK------------------

**NvCaM**  LRHVMTNLGEKLTDEEVDEMIREADIDGDGQVNYEEFVKMMTSK------------------
