# Supplementary material for: An endogenous green fluorescent protein–photoprotein pair in Clytia hemisphaerica eggs shows co-targeting to mitochondria and efficient bioluminescence energy transfer
Source: Open Biol. 2014 Apr 9;4(4):130206. doi: 10.1098/rsob.130206 (PMC4043110; doi:10.1098/rsob.130206)
Supplement: ESM Table 1 [file rsob130206supp2.doc]

**Electronic Supplemental Material (Fourrage et al)**

**ESM Table 1:**

**________________________________________________________________**

**Name Sequence**

**________________________________________________________________**

GFP1F TTGCTGTCCGAATAGTGCAG

GFP1R GACAACTCCTCCTCCGAGTG

GFP2F ACCACCGCACATCATCTACA

GFP2R ATCCTCTTCCCAGGCTCCTA

GFP3F AAAGACGGGGGTTACCAATC

GFP3R TGAAGTGGCTGACTTTGGTG

GFP4F CAAGCTTTCGGAGACGGTAG

GFP4R GATGTGTTCCCTCGTTTCGT

EF1alphaF TGCTGTTGTCCCAATCTCTG

EF1alphaR AAGACGGAGTGGTTTGGATG

Clytin1F AAAAACTTGGCCAAACACGAC

Clytin1R CAATCTAGGCAATTTACAGCAAACA

Clytin2F AAAAGTACTTTAATCCGCGATT

Clytin2R AAAATTTACAATTTCATCTAATATAACAGTAAC

Clytin3F AAACGACTTGCTACCAACGATTT

Clytin3R GTTTCCGTAGAGTCCGTCTGC

**________________________________________________________________**
