# Supplementary material for: An endogenous green fluorescent protein–photoprotein pair in Clytia hemisphaerica eggs shows co-targeting to mitochondria and efficient bioluminescence energy transfer
Source: Open Biol. 2014 Apr 9;4(4):130206. doi: 10.1098/rsob.130206 (PMC4043110; doi:10.1098/rsob.130206)
Supplement: ESM Table 2 [file rsob130206supp3.doc]

**Electronic Supplemental Material (Fourrage et al)**

**ESM Table 2**

**__________________________________________________________________________________**

**Gene family Animal group Species Gene abbreviation Accession N°**

**__________________________________________________________________________________________**

**GFP** Cnidarians- Hydrozoans *Clytia hemisphaerica* CheGFP1 HQ397706

CheGFP2 HQ397707

CheGFP3 HQ397708

CheGFP4 HQ397709

*Clytia gregarium* CgreGFP ADI71927.1

*Phialidium species* PhiYFP AAR85349.1

*Aequorea victoria* AvicGFP CAA58789.1

*Aequorea macrodactyla* AmacGFP AAL33918.1

unclassified anthomedusa AntGFP1 AAR85350.1

AntGFP2 AAR85351.1

Cnidarians- Anthozoans *Renilla reniformis* RrenGFP AAK54757.1

*Nematostella vectensis* NvXP1 XP_001633713.1

NvXP2 XP_001634522.1

*Acropora millepora* AmilGFP AAU06846.1

AmilRFP AAU06852.1

AmilCFP AAU06849.1

*Discosoma species* DisRFP ABC68474.1

*Montastraea cavernos* McavGFP AAU04448.1

McavCFP ABS87206.1

Arthopods- Copepods *Pontellina plumata* PpluGFP1 AY268071.1

PpluGFP2 AY268072.1

*Labidocera aestiva* LaesGFP AY268073.1

Cephalochordates *Branchiostoma floridae* BfGFPa4 XP_002603104.1

BfGFPc1 XP_002601088.1

**Aequorin** Cnidarians- Hydrozoans *Clytia hemisphaerica* CheClyt1 HQ397710

CheClyt2 HQ397711

CheClyt3 HQ397712

*Clytia gregarium* CgreClyt1 BAG49090.1

CgreClyt2 BAG49088.1

*Obelia longissima* OlonObe Q27709.1

*Mitrocoma cellularia* McelMitro P39047.1

*Aequorea victoria* AvicAeq2 AAA27717.1

AvicApo BAC81730.1

AvicAeq AAT11852.1

*Aequorea macrodactyla* AmacApo AAK02061.1

*Aequorea coerulescens* AcoeAeq AAO91813.1

**Calmodulin**  Cnidarians- Hydrozoans *Clytia hemisphaerica* CheCaM AAY16242.1

*Clytia gracilis* CgraCaM AAY16241.1

*Obelia longissima* OlonCaM AAY16245.1

Cnidarians- Anthozoans *Nematostella vectensis* NvCaM XP_001638581.1 *Renilla reniformis* RrenLBP P05938.1

RrenCaM P62184.2

__________________________________________________________________________________
